# A Mixed‐Methods Study Exploring the Feasibility of a Digital Combined Lifestyle Intervention for Patients With Post Covid‐19 Condition

**DOI:** 10.1111/hex.70299

**Published:** 2025-05-25

**Authors:** Debbie Gach, Charlotte D. C. Born, Lisanne L. T. Schuurman, Frits H. M. van Osch, Joop P. van den Bergh, Sanne M. P. L. Gerards, Rik Crutzen, Annemie M. W. J. Schols, Rosanne J. H. C. G. Beijers, the P4O2 Consortium

**Affiliations:** ^1^ Department of Respiratory Medicine, NUTRIM, Institute of Nutrition and Translational Research in Metabolism Maastricht University Medical Centre+ Maastricht the Netherlands; ^2^ Department of Clinical Epidemiology VieCuri Medical Centre Venlo the Netherlands; ^3^ Department of Epidemiology, GROW, Research Institute for Oncology and Reproduction Maastricht University Medical Centre+ Maastricht the Netherlands; ^4^ Department of Internal Medicine VieCuri Medical Centre Venlo the Netherlands; ^5^ Department of Internal Medicine, NUTRIM, Institute of Nutrition and Translational Research in Metabolism Maastricht University Medical Centre+ Maastricht the Netherlands; ^6^ Department of Health Promotion, NUTRIM, Institute of Nutrition and Translational Research in Metabolism Maastricht University Medical Centre+ Maastricht the Netherlands; ^7^ Department of Health Promotion, CAPHRI, Care and Public Health Research Institute Maastricht University Medical Centre+ Maastricht the Netherlands

**Keywords:** diet, dietary supplements, digital health, healthy lifestyle, physical activity, post‐Covid‐19 condition, proof‐of‐concept study

## Abstract

**Introduction:**

Low physical activity and poor dietary quality can negatively influence Covid‐19 recovery and increase the risk and duration of post‐Covid‐19 condition (PCC). This proof‐of‐concept nested intervention study aimed to evaluate the feasibility of a digital personalised combined lifestyle intervention (CLI) in patients with PCC using a mixed‐methods design, assessing compliance, experiences and perceived effectiveness.

**Methods:**

A nested intervention study, incorporating motivational interviewing aiming to enhance physical activity and dietary quality, was conducted within a multicentre prospective cohort study including 95 post‐Covid‐19 patients (aged 40–60) between May 2021 and September 2022. Patients in the intervention and control groups were followed at ±3–6 and ±12–15 months post Covid‐19. The intervention consisted of nine monthly individual counselling sessions (30 min), two interactive‐group sessions (60 min), and three educative webinars (45 min). Additionally, a nutritional supplement (NS; Remune, Smartfish, Oslo, Norway) high in omega‐3 fatty acids, vitamin D and protein was provided to facilitate recovery. After the intervention, a process evaluation was conducted, comprising an evaluation questionnaire and semi‐structured in‐depth interviews.

**Results:**

The intervention‐to‐treat group consisted of 47 patients (age 54.7 ± 6.0 years; 40% males; BMI 30.6 ± 5.8 kg/m^2^) of whom 74% had ≥ 8 individual sessions via telephone (66%) or video call (34%). Over half of the group (55%) attended the educative webinars, while attendance was lower in the interactive‐group sessions, with 32% attending one session and 15% two sessions. The process evaluation indicated that patients were satisfied with the digital coaching and the frequency, duration and content of the sessions. Half of the patients reported perceived improvements in physical activity levels and dietary quality throughout the intervention, with the majority also reporting sustainment of these lifestyle changes post‐intervention.

**Conclusion:**

A digital personalised CLI was well‐received among patients with PCC regarding compliance, experiences and perceived effectiveness. These findings will guide the development and implementation of tailored interventions to enhance overall well‐being among patients with PCC.

**Patient or Public Contribution:**

Patients' experiences regarding the design and implementation of the study were retrieved. Although participants were not directly involved in the initial design of the study, their experiences were actively incorporated into the refinement and implementation of the study procedures, thereby ensuring meaningful patient involvement.

## 1 Introduction

Post‐Covid‐19 condition (PCC) represents a complex and multifaceted global health challenge, with 13% of the people experiencing symptoms longer than 3 months after the acute SARS‐CoV‐2 infection [1, 2]. PCC has an impact on multiple organ systems, which manifests in a wide spectrum of symptoms including persistent fatigue, dyspnoea, chest pain, smell and taste dysfunction, painful muscles and cognitive impairments [1, 3]. Interestingly, PCC has been observed in diverse Covid‐19 cases ranging from mild to severe acute disease severities [4].

A lifestyle characterised by low physical activity and a poor dietary quality can negatively influence Covid‐19 recovery and increase the risk and duration of PCC [5, 6, 7]. Patients with PCC generally show reduced physically active levels compared to pre‐Covid‐19, which has been linked to poorer psychological health, resulting in lower self‐esteem, frustration and feelings of guilt [8, 9]. Furthermore, patients with PCC who remained active experienced shorter PCC duration and symptom relief, indicating a protective effect of physical activity [7]. Besides physical inactivity, an unhealthy diet, high in saturated fats, red and processed meats, and refined grains, alongside insufficient intake of fruits, vegetables, whole grains and fish, has been associated with an increased risk of PCC [5]. Consumption of higher levels of proteins and lipids, including unsaturated fats and cholesterol, before infection has already been demonstrated to positively impact recovery following an acute SARS‐CoV‐2 infection [6]. In general, a healthy diet consisting of adequate vitamins (e.g., vitamins A and D), minerals (e.g., zinc and iron), probiotics and sufficient energy and protein intake is recommended in PCC management [10, 11]. Specifically, vitamin D and omega‐3 polyunsaturated fatty acids (PUFAs) may mitigate the persistent inflammatory response and immune dysregulation observed in these patients due to their immunomodulatory and anti‐inflammatory properties [11, 12, 13]. Additionally, adequate protein intake is crucial for the restoration of muscle mass and strength lost during acute Covid‐19 [11].

Combining physical activity and nutritional interventions may synergistically enhance PCC recovery [14]. Sustained long‐term lifestyle changes and optimal recovery outcomes require behaviour modification, which could be achieved with counselling strategies [15]. Counselling based on self‐determination theory (SDT) aims to support individuals in developing greater autonomy, competence and relatedness to achieve a sustainable behavioural change [16]. Motivational interviewing (MI) complements this by empowering active participation in someone's own change process (e.g., through goal setting) and enhancing a person's intrinsic motivation [17]. To date, limited combined lifestyle interventions (CLIs), focusing on both physical activity and diet, have been studied in patients with PCC [18, 19, 20, 21, 22, 23, 24, 25, 26]. The majority were digital interventions due to pandemic restrictions [18, 19, 20, 21, 22, 23]. However, the digital component offers the advantage of wider reach and accessibility as well as patient engagement [27]. Although CLIs seem to have beneficial effects in patients with PCC, current studies were short‐term interventions (between 1 and 3 months) in a controlled setting, primarily focused on adherence to specific regimens, rather than achieving sustainable long‐term lifestyle modifications and recovery [18, 19, 20, 21, 22, 23, 24, 25, 26].

Within the Precision Medicine for more Oxygen COVID‐19 study (P4O2 COVID‐19), a digital personalised CLI was developed that incorporated MI to enhance both physical activity levels and dietary quality among patients with PCC. Additionally, a nutritional supplement (NS) high in protein, vitamin D and omega‐3 PUFAs was provided. The primary aim of this proof‐of‐concept nested intervention study is to evaluate the feasibility of a digital personalised CLI in patients with PCC using a mixed‐methods design, by assessing compliance, experiences and perceived effectiveness. Additionally, changes in health outcomes over time among patients with PCC are described.

## 2 Methods

### 2.1 Study Design and Population

This proof‐of‐concept study was a nested intervention within a multicentre prospective observational cohort study in the Netherlands. To evaluate the feasibility of the intervention, compliance, experiences and perceived effectiveness were analysed using a mixed‐methods concurrent triangulation design. A detailed description of the cohort study has already been described elsewhere [28]. Briefly, patients were recruited from post‐Covid‐19 outpatient clinics in five hospitals in the Netherlands between May 2021 and September 2022. Patients were eligible for inclusion if they were aged between 40 and 60 years, had a proven SARS‐CoV‐2 infection (positive PCR test, proven serology or a CORADS score ≥ 4), the ability to provide written informed consent, access to the internet and understood the Dutch language. Two follow‐up visits took place at ±3–6 and ±12–15 months post‐Covid‐19 infection in parallel to the Covid‐19 outpatient clinic visits. As previously reported by Baalbaki et al., most patients exhibited persistent symptoms, with fatigue observed in 76% and respiratory and neurological complications in 79% and 68%, respectively, at the first study visit. Consequently, our study cohort is classified as patients with PCC [28].

The study was approved by the ethical board of the Amsterdam University Medical Centre (NL74701.018.20). All patients gave written informed consent before enrolling in the study.

### 2.2 Intervention

**Figure 1:**
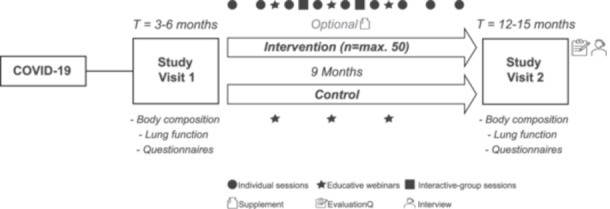
Study design of the digital personalised combined lifestyle intervention study.

All patients were invited to participate in the lifestyle intervention, which included individualised digital counselling sessions, interactive‐group sessions, educative webinars and the optional provision of an NS over a 9‐month follow‐up period (Figure 1). A maximum of 50 patients could be included in the intervention group, with allocation based on voluntary participation. If patients were not willing to participate, they were considered as controls and followed the regular track of the study. When a total of 50 patients was reached in the intervention group, additional patients were directly allocated to the control group. The 9‐month intervention period and the counselling approach, which applied MI, were both based on previous studies [29, 30, 31]. The counselling intervention was executed by two coaches (D.G. and L.S.), who were both trained by a senior health coach with extensive expertise in performing this specific counselling approach.

The monthly individual counselling sessions (30 min) took place via telephone or video call. During the initial intake session (45–60 min), patients were asked to construct personal learning goals based on their physical activity level and dietary quality, which they aimed to accomplish in the following month. Every month, the same coach (D.G. or L.S.) tracked the progression of the learning goals using MI. Additionally, two digital interactive‐group sessions (60 min) were offered during which patients were stimulated to discuss personal learning goals, share experiences and exchange tips or strategies. Furthermore, three digital educative webinars (45 min) took place: (1) Covid‐19 experiences from the physician's point of view, (2) rehabilitation trajectory after a Covid‐19 infection and (3) the role of nutrition in the recovery process after a Covid‐19 infection. During these sessions, experts informed the patients with relevant insights and provided them with useful tips to improve their physical activity and dietary quality. A recording link of the session was sent to patients who were not able to attend the live sessions. The information gained during the interactive‐group sessions and educative webinars and its applicability were discussed for each patient during the individual sessions. Patients who were not included in the intervention (i.e., control group) were also simultaneously invited to attend the educative webinars. Lastly, as part of the cohort study, both groups received a GARMIN Vivosmart 4 activity tracker to monitor physical activity (e.g., steps and intensity), heart rate and sleep quality.

### 2.3 NS

The intervention group was offered an additional NS ( ± 230 kcal, Remune, Smartfish, Oslo, Norway), containing omega‐3 PUFAs (2.0 g) including eicosapentaenoic acid (EPA; 0.8 g) and docosahexaenoic acid (DHA; 1.2 g) from fish oils, 25‐hydroxyvitamin D3 (10 µg) and whey protein concentrate (10 g). The NS was available in peach and raspberry flavours, and patients could choose or alternate between them to increase compliance. In general, patients were advised to consume one 200‐mL supplement each day. Only in the case of malnutrition, defined by the GLIM criteria, patients were advised to take two supplements per day [32].

### 2.4 Data Collection

#### 2.4.1 Process Evaluation

##### 2.4.1.1 Evaluation Questionnaire—Quantitative

After completion of the intervention (ranging between 1 and 13 months), all patients were asked to complete an evaluation questionnaire (evaluationQ) to evaluate the perceived effectiveness of the lifestyle intervention and patients' experiences with the programme. The evaluationQ was specifically developed for this study, in collaboration with post COVID‐researchers, including qualitative research specialists, and consisted of 55 statements regarding reasons to participate, patients' experiences (overall and concerning the different sessions), goals on physical activity and dietary quality, and lifestyle factors (motivation regarding lifestyle, lifestyle changes during the intervention and continuation of lifestyle changes after the intervention). The evaluationQ was completed on paper, and patients had to select the best fitting statement out of a minimum of two and a maximum of four answer options.

##### 2.4.1.2 Semi‐Structured In‐Depth Interviews—Qualitative

A subgroup was also invited to participate in semi‐structured in‐depth interviews, which applied a descriptive phenomenological method to gain deeper insights into the perceived effectiveness of the intervention and patients' lived experiences [33]. An interview topic guide was developed, partly based on questions and answers given in the evaluationQ, to structure the interviews. The interview topic guide covered open questions related to two main constructs: experiences with the intervention and perceived effects of the intervention. Two researchers (D.G. and C.B.) conducted the interviews. After conducting the first two interviews, the researchers reviewed their performance with a qualitative research specialist to receive feedback and improve the execution of subsequent interviews. Since D.G. was also involved in the implementation of the lifestyle intervention, she did not interview patients she had personally counselled, to prevent bias and preserve independence. A balanced selection of patients was asked to participate in the interviews, considering gender and timing of finishing the intervention. The interviews were conducted via telephone or video call, depending on the patient's preference. All interviews were in Dutch and audio recorded. Researchers (D.G. and C.B.) had regular meetings to discuss the level of saturation, which was reached when no new information emerged. Subsequently, no additional interviews were conducted.

#### 2.4.2 Health Outcomes Study Visits

Baseline and hospitalisation characteristics were collected during the first study visit. At both study visits, body composition was assessed by bioelectrical impedance analysis (BIA; Bodystat 500, EuroMix, Leuven, Belgium), blood samples were collected to evaluate compliance with the NS by analysing fatty acid (FA) profiles, and several questionnaires were administered and completed by the patients. Pulmonary function tests were performed during the Covid‐19 outpatient clinic visits. A detailed description of the health outcomes assessment procedures can be found in Supporting Information 1.

### 2.5 Data Processing and Analysis

For comparison of patient characteristics between the intervention and control group, a *χ* ^2^ test was calculated and an independent samples *t*‐test or Mann–Whitney *U* test as appropriate. Descriptive statistics were used to analyse the compliance with the lifestyle intervention and NS. The evaluationQ was analysed using descriptive statistics, and responses were reported as absolute frequencies with corresponding percentages. To evaluate changes in health outcomes between study visits 1 and 2 in the intervention and control group, a paired samples *t*‐test or Wilcoxon signed rank test was calculated as appropriate for continuous variables and a McNemar test for ordinal/categorical variables. All analyses were performed using IBM SPSS statistics, version 28. A *p* value < 0.05 was considered statistically significant.

Qualitative data were transcribed verbatim using an online tool (Amberscript Global B.V., Amsterdam, The Netherlands). Afterwards, the researchers checked and adjusted the transcripts. Coding of the interviews was done using Atlas.ti‐23 software. A predominantly deductive research approach was applied as two pre‐established coding trees were used to analyse the interviews (Supporting Information 2; Figure S1). Framework analysis was applied, and two main themes were defined from the beginning: 1. Experiences with the intervention and 2. Perceived effects of the intervention. The first main theme was divided into three pre‐defined sub‐themes: 1.1. Reasons for participation, 1.2. General experience and 1.3. Programme delivery. The second main theme was divided into the following five pre‐defined sub‐themes: 2.1. Lifestyle in general, 2.2. Lifestyle during the intervention, 2.3. Lifestyle after the intervention, 2.4. Emotional well‐being and 2.5. Health status. Two researchers (D.G. and C.B.) independently coded interviews through weekly consensus meetings to discuss discrepancies, establish several decision rules, and modify the coding trees as appropriate. After five interviews were coded, 73% agreement was reached between the two researchers; therefore, D.G. analysed the remaining eight interviews (62%). All interviews were re‐evaluated using the finalised coding framework, and a conclusive meeting was conducted to address any remaining uncertainties in the analyses.

## 3 Results

### 3.1 Patient Characteristics

**Figure 2:**
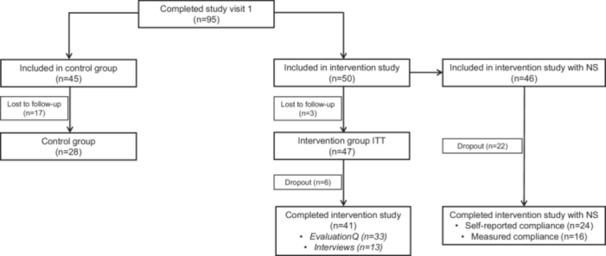
Flow chart of the (included) study population. EvaluationQ, evaluation questionnaire; ITT, intention to treat; NS, nutritional supplement.

In total, 50 patients were included in the intervention, of which three patients were lost to follow‐up, resulting in an intervention‐to treat (ITT) group of 47 patients (Figure 2). Eventually, 41 patients completed the full intervention study, of whom 24 patients had NS based on their self‐reported compliance. After completion of the intervention, 33 patients completed the evaluationQ (*n* = 4 dropouts and *n* = 4 lost to follow‐up), and 13 interviews were performed. Patients who were not included in the intervention were considered as controls (*n* = 28).

Patients in the intervention group had a mean age of 54.7 ± 6.0 years, 40% were male and mean BMI was 30.6 ± 5.8 kg/m^2^, with half of the patients being obese (51%; Table 1). Of the 47 patients, 42 (89%) were admitted to the hospital, of whom 13 (28%) were also admitted to the intensive care unit (ICU). No differences in patient characteristics were observed between the intervention and control groups.

**Table 1:** Patient characteristics of the intervention and control group.

Patient characteristics			
	Intervention (*n* = 47)	Control (*n* = 28)	*p* value
Age in years	54.7 ± 6.0	54.1 ± 6.2	0.704
Male	19 (40)	17 (61)	0.089
Ethnicity^b^			0.645
White	37 (82)	21 (78)	
Other	8 (18)	6 (22)	
Smoking status			0.154
Current	0 (0)	2 (7)	
Ex	24 (51)	15 (54)	
Never	23 (49)	11 (39)	
BMI in kg/m^2^	30.6 ± 5.8	29.6 ± 4.9	0.472
BMI categories^c^			0.627
Normal weight (18.5–25.0 kg/m^2^)	5 (11)	5 (19)	
Overweight (25.0–30.0 kg/m^2^)	18 (38)	9 (33)	
Obese (≥ 30.0 kg/m^2^)	24 (51)	13 (48)	
Comorbidities			
Asthma	9 (19)	6 (21)	0.726
COPD	4 (9)	1 (4)	0.513
Cardiovascular disease	9 (19)	10 (36)	0.177
Heart failure	2 (4)	4 (14)	0.232
Diabetes	6 (13)	5 (18)	0.630
Renal failure	1 (2)	3 (11)	0.277
Hospitalisation	42 (89)	25 (89)	≥ 0.999
Hospital stay in days^a^	8 (4–16)	9 (3–15)	0.908
ICU admission	13 (28)	8 (29)	0.932
Days between infection and study visit 1	167 ± 36	173 ± 34	0.442
Days between infection and study visit 2	472 ± 54	468 ± 47	0.728

*Note:* Data are shown as mean ± SD or *n* (%) unless indicated otherwise.

Abbreviations: BMI, body mass index; COPD, chronic obstructive pulmonary disease; ICU, intensive care unit.

^a^ Median (IQR).

^b^ Assessed in 45/27 of the intervention/control group.

^c^ Assessed in 47/27 of the intervention/control group.

### 3.2 Compliance of the Lifestyle Intervention and NS

Most patients performed more than eight individual counselling sessions (74%) via telephone (66%) or video call (34%) (Table 2). One‐third of the patients (32%) took part in one interactive‐group session and 15% in two interactive‐group sessions during the intervention. The educative webinars on ‘COVID‐19 experiences from a physician’ and ‘Rehab trajectory after COVID‐19’ were attended by more than half of the group (58%), live (30% and 28%, respectively) or viewed afterwards via the recording link (28% and 30%, respectively). Half of the group (51%) attended the session on ‘The role of nutrition in COVID‐19 recovery’, 28% live and 23% via the recording link.

**Table 2:** Compliance of the lifestyle intervention sessions.

Compliance of the lifestyle intervention	Intervention (*n* = 47)
Communication medium	
Telephone	31 (66)
Video call	16 (34)
Individual counselling sessions performed	
1–3 session(s)	4 (9)
4–7 sessions	8 (17)
8–9 sessions	35 (74)
Interactive‐group sessions attended	
0 sessions	25 (53)
1 session	15 (32)
2 sessions	7 (15)
Educative webinars attended	
1. Covid‐19 experiences from a physician	27 (58)
− Live	14 (30)
− Recording link	13 (28)
2. Rehab trajectory after Covid‐19	27 (58)
− Live	13 (28)
− Recording link	14 (30)
3. Role of nutrition in Covid‐19 recovery	24 (51)
− Live	13 (28)
− Recording link	11 (23)

*Note:* Data are shown as *n* (%).

**Figure 3:**
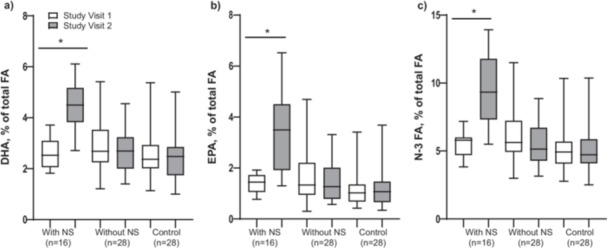
Plasma levels of the supplemented nutrients DHA (a), EPA (b) and *N*‐3 FA (c). Note**:** Data are shown as median (IQR). *Significantly different from visit 1, *p* < 0.05. DHA, docosahexaenoic acid; EPA, eicosapentaenoic acid; FA, fatty acid; NS, nutritional supplement.

Compliance with the NS was established through EPA, DHA and *N*‐3 fatty acid (FA) plasma levels. Twenty‐two patients prematurely stopped using the NS during the intervention and were allocated to the intervention group without NS for the analysis. Additionally, from the 24 patients that completed the intervention with NS based on their self‐reported compliance, seven patients showed a decline in DHA, EPA and *N*‐3 FA levels between study visits 1 and 2, and these were therefore considered non‐compliant and analysed in the intervention group without NS. Furthermore, two patients were excluded from the analysis because of missing blood sampling data at study visit 2. Plasma levels of DHA, EPA and *N*‐3 FA increased in the intervention group with NS (*p* < 0.001), but not in the intervention group without NS and control group (*p* > 0.05; Figure 3a,b,c). In all three groups, improvements were seen over time for FFMI, while pulmonary function outcomes (FEV_1_, FVC and DLCO) only increased in the intervention group with NS (*p* < 0.05; Table S1; Supporting Information 3).

### 3.3 Process Evaluation

The 33 patients who performed the process evaluation with evaluationQ and the 13 patients who completed the evaluationQ as well as the interview were comparable to the total intervention group (*n* = 47) as shown in Table S2 (Supporting Information 4). Results of the evaluationQ are described in Table S3 (Supporting Information 5) and further discussed below alongside the findings of the semi‐structured in‐depth interviews.

#### 3.3.1 Experiences With the Intervention

##### 3.3.1.1 Reasons for Participation

The main reasons for participation in the intervention as reported by patients were that they wanted to *become healthier* and *improve their physical conditioning* (each 49%). Patients specifically mentioned improving their own health to recover from PCC as fast as possible.

‘It's also for the sake of my own health, because I participated for myself, just buckling down and giving my best to get better.’

Male (Participant A)

Some patients also indicated that they found it important to participate in the intervention to expand our understanding of this new disease condition, given the substantial gaps in existing knowledge. Patients hoped that the expanded knowledge would benefit future people encountering PCC, by preventing them from enduring the same suffering as they had experienced.

‘I found it really important to participate, so you could get more information about the misery which I have been through, and hopefully this can further be applied to other people who will experience the same after me.’

Male (Participant B)

##### 3.3.1.2 General Experience

In general, patients were really satisfied with their experience of participating in the intervention. All patients reported to have been *satisfied* with the functioning of the coaches during the lifestyle intervention, and most reported that they were also *satisfied* with the course of the intervention (94%). More specifically, patients liked the intensive contact with the coaches and found the way of contact to be pleasant and safe, largely due to the empathetic support of the coaches. Additionally, patients appreciated the personalised approach of the intervention, as well as the good organisation of the different sessions during the intervention.

‘What I really liked was the way she (the coach) approached me every time and the interaction that we had. I found it really pleasant, and she was very friendly and supportive.’

Male (Participant C)

One patient was less satisfied with the timing of the intervention, as she felt that it was too early for her because she was still in the process of recovering from Covid‐19. Consequently, she felt unable to fully focus and participate effectively in the intervention.

‘Maybe it was too early for me when I started with it (the intervention), as I was still busy recovering for myself from the disease.’

Female (Participant D)

All patients also reported being satisfied with the communication of the coaches during the intervention, and the majority reported that the intervention went as expected based on the information they obtained (97%). Patients found the communication very clear, particularly the well‐arranged appointments by the coaches. The active and open communication was also valued, contributing to the opportunities for patients to ask questions and receive direct responses. Additionally, patients indicated that they had sufficient information about what the intervention entailed, with the information being clearly provided and extensively explained by the coaches. Consequently, the clear communication and thorough explanation of the provided information ensured that the expectations of the patients aligned with the reality and that they knew what to expect from the intervention.

‘What the sessions included was well communicated, you did know what to expect beforehand.’

Male (Participant A)

Some patients, however, indicated that they expected more coaching guidance on nutrition from the coaches, such as specific diet‐related tips that could enhance their recovery process. One patient also indicated that she expected more answers to questions related to her Covid‐19 complaints from the coaches.

‘Although I read all the information beforehand, I also expected a little bit that you would answer questions related to COVID‐19 complaints. But after the first session, I heard that this was not part of your study and that was maybe the only thing that I missed a little bit.’

Female (Participant E)

##### 3.3.1.3 Programme Delivery

More than 85% of the patients indicated that they considered the three different types of sessions *useful*. The individual counselling sessions were found useful because of the interactive approach used, the motivational and emotional support received from the coaches, and the personalised content related to diet and physical activity that was provided. Patients also found the healthy lifestyle‐related tips from the coaches useful, as well as the process of setting and evaluating goals every month, as they felt that this contributed to a greater awareness and knowledge of healthy lifestyle habits.

‘What really helped is if you have someone like her (the coach), who thinks with you and who motivates and stimulates and guides you. “How can you approach this?” That really helped me. And what I just said about walking, that I thought I had to walk every day and that she (the coach) said for example, but if you try every other day, than you still make it to three or four times per week and then the chances are higher that you will maintain and keep your motivation. Plus, you don't exceed your borders, so you don't get setbacks. In that way, we looked at what was achievable.’

Female (Participant F)

Most patients reported finding the interactive‐group sessions useful due to the opportunity to share experiences with others on topics such as PCC‐related complaints, encountered obstacles and achieved milestones in recovery, and healthy lifestyle tips. This resulted in a positive and educative experience in a safe and trusted environment for most patients. However, some patients noted that the interactive‐group sessions were not useful, as they lacked constructive tips and were dominated by nagging about complaints, which created a negative ambience. Additionally, some patients expressed feelings of guilt when their overall well‐being was better than that of others, making them hesitant to participate in subsequent sessions.

‘Everyone was just moaning about what they experienced, oh I feel like this and oh I feel like that, but there was no real contact and support between each other. I did not find it constructive at all.’

Female (Participant G)

Although more than half of the patients indicated that a lot of the information given during the educative webinars was already known to them, the sessions were still perceived as beneficial by most patients. Patients mentioned that certain information was already obtained through self‐research, news sources or their treating physicians. The content on the ‘COVID‐19 experiences from a physician’ was particularly valued for enhancing understanding and acceptance of certain treatment decisions that were made during the patient's hospitalisation for Covid‐19. Additionally, other content related to tips around optimising recovery (i.e., the implementation of pacing strategies), practical advice on a healthy diet and the importance of protein in muscle restoration after illness were mentioned as useful information by patients. Nevertheless, some feedback was provided for areas to improve, such as implementing a more interactive approach, including content on mental health problems and providing more tips on recovery strategies.

‘So, I thought, dairy products contain proteins which are needed for your muscles. I did not know that, but these are things that I learned from it (the third educative webinar), that I have to take extra of these to rebuild my muscles.’

Female (Participant H)

The overall duration of the intervention was received positively by most patients, who acknowledged the importance of the 9‐month period for achieving long‐term healthy lifestyle effects and research purposes. Most patients appreciated the frequency of the sessions, as well as their even distribution, which made it manageable alongside other life commitments such as work, family and health appointments. Although most patients were content with the timing and duration of the interactive group sessions and educative webinars, some found the sessions too late in the day and the duration too long due to persisting concentration problems and fatigue, making participation challenging for them.

‘If I have to think deeply and concentrate, then I can be happy if I manage it for 15 min or half an hour and that is okay. This is of course because of COVID‐19, so yeah, in my case, a shorter session would be more welcome.’

Male (Participant A)

Most patients mentioned having had a positive experience with the digital communication medium and found it a valuable tool to easily reach a lot of people across a wide area. However, a small number of patients reported to have experienced *problems* with their digital connection during the educative webinars (22%) or the interactive‐group sessions (6%). Additionally, some patients indicated a preference for in‐person sessions, especially during the interactive‐group sessions when meeting other participants and when discussing personal stuff within the individual sessions.

‘Of course, it would be nicer if it was not all done digitally. That you would have met one time in‐person would have been nicer, especially with a group.’

Female (Participant I)

#### 3.3.2 Perceived Effects of the Intervention

##### 3.3.2.1 Lifestyle in General

Most patients reported that their lifestyle *improved* (64%) throughout the intervention. Furthermore, almost all patients reported that the intervention resulted in a greater awareness and knowledge of healthy lifestyle habits (97%). Specifically, the monthly coaching in the individual counselling sessions and the content of the educative webinars made them more aware of the characteristics of a healthy lifestyle, as well as the importance of pursuing a healthy lifestyle to improve their recovery.

‘It (the intervention) made me more aware that I am not going to feel better by eating a bag of chips and that I will feel much better by going for a run and that this has many more benefits.’

Female (Participant J)

Almost all patients also found it important and were motivated to improve their lifestyle during the intervention. The main factors for the high motivation were participation in the lifestyle intervention as well as the desire to recover from PCC. Moreover, patients indicated that the support of the coaches during the individual counselling sessions, as well as the opportunity to set and work independently on their lifestyle goals, enhanced their motivation even more. Some patients, however, indicated that their motivation dropped during the intervention due to periods of not feeling well and when stagnations occurred in their recovery process.

‘I was really motivated to become the old me, because I thought, well, I have to live, I have a family, I still want to do a lot of things. It is also pleasant if I can fully live my life again.’

Female (Participant F)

##### 3.3.2.2 Lifestyle During Intervention

Half of the patients reported having *increased* their physical activity levels during the intervention, which was mainly achieved by cycling and walking more. Moreover, most patients (82%) reported having reached their physical activity goals, either *independently* (58%) or *with help from the coach* (24%) during the intervention. The positive guidance of the coaches and their tips on how to gradually increase physical activity levels were mentioned by patients as factors for achieving their goals. Additionally, setting goals and rewards for achieving goals, tracking physical activity with a smartwatch, receiving support from loved ones and pulling back on other activities were other stimulating factors reported by patients for improved physical activity levels. One patient, however, indicated that PCC complaints still limited his ability to engage in physical activity, and another patient mentioned being busy working again as a limiting factor for not reaching her goals.

‘I just started cycling very short distances. But every time it was a victory, if I could see after a week that I cycled 500 meters further…. And I felt that she (the coach) really pulled me through, because she was so positive which gave me an extra push, like, you know, you can do this and there is someone that supports you, someone else, outside your own family.’

Female (Participant K)

In line with the physical activity goals, half of the patients reported eating healthier during the intervention, of whom around 70% attributed *eating healthier* to the intervention. Overall, most patients reported having reached their dietary intake goals throughout the intervention, *independently* (55%) or *with help from the coaches* (27%). For dietary intake, the main goal setting was related to increasing protein and water intake, eating more fresh ingredients (fruits and vegetables), smaller portion sizes and decreasing consumption of candy and soft drinks. One patient also mentioned cooking more for herself as a goal, but this remained challenging as she still encountered decreased energy levels due to the PCC. Mainly, the process of goal setting and repeating/evaluating dietary intake goals every month with the coach was mentioned as a stimulating factor by patients for pursuing a healthier diet. In addition, patients mentioned that the independence to construct their own lifestyle goals resulted in a greater accomplishment of achieving a healthier lifestyle, as they could choose for themselves what they found important to work on.

‘Setting goals and then repeating it every time that you had an appointment. And then evaluating, how did it work out or not? And then I thought, yeah, I sustained my diet for at least 3 days every week, which went better than last month…That was really a reminder every month, you get more aware, although you know it, it just made you more aware again.’

Female (Participant J)

Overall, the perceived health effects of the NS were insufficient for most patients, with some also expressing feelings of disappointment as they had higher expectations of its efficacy. Furthermore, some patients prematurely stopped with the NS throughout the intervention, with the main reasons being dissatisfaction with the taste, the high‐caloric density counteracting their weight loss goals and the occurrence of adverse effects such as nausea.

‘When I started my diet, we (the coach and I) talked about it (the NS), because I used them. And then we (the coach and I) decided that I would stop consuming them, because of the high calories that are in it.’

Female (Participant F)

##### 3.3.2.3 Lifestyle After Intervention

Most patients had the intention and were motivated to continue with their lifestyle changes after the intervention, as they acknowledged the importance of a healthy lifestyle for their overall well‐being and recovery. One patient, however, indicated that his motivation had dropped since he experienced smaller increments in his progress.

‘Recently, my motivation is far less, because the big improvements that I made in the beginning, I don't see them as big steps anymore.’

Male (Participant B)

Overall, patients mentioned having succeeded in continuing their lifestyle changes after the intervention and that the intervention resulted in the implementation of the new lifestyle habits into their daily routine. One patient also indicated that the intervention helped her to lay the fundamentals again for a healthy life balance, considering not only lifestyle but also work and family responsibilities. Some patients, however, indicated that they could only sustain the healthy lifestyle habits for a certain period, and the biggest challenge was to stay consistent. Common pitfalls leading to relapsing into old routines were increments in the busyness of daily life (i.e., family and work), setbacks in disease recovery and bad weather circumstances. Some patients also still struggled with physical activities such as walking up stairs and higher‐intensity exercises due to ongoing PCC fatigue.

‘It (the intervention) helped to make choices on what you find important. And I think that it is good that at that moment, I choose to go for an‐hour walk, because I have the need to go outside and anyhow, I love that.’

Female (Participant F)

‘Recently, it (the lifestyle changes) went well, I totally got it. But then when I am not feeling good, when I get sick again, I totally lose it.’

Female (Participant L)

##### 3.3.2.4 Emotional Well‐Being

All patients appreciated the emotional support from the coaches throughout the intervention, and some felt that this also positively impacted their mental health status. Especially, the opportunity to share their PCC experiences and complaints with people who have a better understanding and acceptance of the condition was found useful and pleasant by patients. Patients also reported that the interaction with other patients during the interactive‐group sessions supported them mentally, as they realised they were not alone in their experiences.

‘There was a lot of misunderstanding back then around PCC, because you did not see it necessarily from the outside, so there was nothing going on. And, with her (the coach), it was always pleasant, and I did not feel judged.’

Female (Participant G)

##### 3.3.2.5 Health Status

A small number of patients indicated to be fully recovered from the PCC. Despite ongoing symptoms in some patients, nearly all were satisfied with their recovery progress during and after the intervention. The most common persisting symptoms included fatigue, memory and concentration problems, shortness of breath and reduced physical conditioning. Consequently, patients found tasks more time‐consuming and required more rest than before, which led to occasional frustration. Nonetheless, some patients accepted the current situation and remained optimistic about achieving full recovery soon.

‘I can do a lot more, but unfortunately not everything that I could before the COVID‐19. Because everything goes a lot slower nowadays.’

Male (Participant B)

### 3.4 Health Outcomes Study Visits

For body composition outcomes, FFMI increased in both the intervention and control groups between visits 1 and 2 (*p* = 0.003 and *p* = 0.026, respectively; Table 3). Within the intervention group, FEV_1_, FVC and DLCO increased over time (*p* < 0.05), while no changes were observed in the control group (*p* > 0.05). EQ‐5D scores did not change over time in both groups (*p* > 0.05). Decrements in impaired FSS scores were seen over time in the intervention (*p* = 0.039) but not in the control group (*p* = 0.219). Patients in the intervention showed high levels of intrinsic motivation and identified regulation for pursuing healthy eating and physical activity behaviour at both study visits.

**Table 3:** Health outcomes of the intervention and control group at study visits 1 and 2.

	Intervention (*n* = 47)		Control (*n* = 28)	
Health outcomes study visits	Visit 1	Visit 2	Visit 1	Visit 2
Body composition^b^				
BMI in kg/m^2^	30.5 ± 5.4	30.9 ± 5.5	30.0 ± 4.4	30.4 ± 4.4
FFMI in kg/m^2^	19.5 ± 2.5	20.1 ± 2.6*	19.7 ± 1.9	20.4 ± 2.1*
FMI in kg/m^2^	11.0 ± 4.0	10.8 ± 4.3	10.3 ± 3.7	9.9 ± 3.6
Pulmonary function^c^				
FEV_1_ in %pred	83.1 ± 19.8	88.2 ± 15.9*	83.0 ± 13.8	85.7 ± 14.0
FVC in %pred	83.3 ± 22.5	89.6 ± 17.5*	82.1 ± 13.7	84.7 ± 11.3
FEV_1_/FVC in %	98.1 ± 15.8	101.2 ± 21.3	100.4 ± 13.5	99.5 ± 13.2
DLCO in %pred	75.2 ± 20.1	83.1 ± 15.6*	65.8 ± 16.9	66.5 ± 16.8
Questionnaires				
EQ‐5D score^a^^,^ d	0.76 (0.58–0.89)	0.85 (0.70–0.91)	0.85 (0.68–1.00)	0.89 (0.68–1.00)
EQ‐5D categories^a^^,^ d				
Mobility	1.00 (1.00–3.00)	1.00 (1.00–2.00)	1.00 (1.00–2.00)	1.00 (1.00–3.00)
Self‐Care	1.00 (1.00–1.00)	1.00 (1.00–1.00)	1.00 (1.00–1.00)	1.00 (1.00–1.00)
Usual activities	3.00 (1.00–3.00)	2.00 (1.00–3.00)	2.00 (1.00–3.00)	1.00 (1.00–2.00)
Pain/discomfort	2.00 (1.00–3.00)	1.50 (1.00–3.00)	1.00 (1.00–2.00)	2.00 (1.00–3.00)
Anxiety/depression	1.00 (1.00–2.00)	1.00 (1.00–2.00)	1.00 (1.00–2.00)	1.00 (1.00–1.00)
HADS anxiety > 10^e^	2 (5)	2 (5)	2 (10)	3 (14)
HADS depression > 10^e^	3 (8)	1 (3)	1 (5)	2 (10)
FSS ≥ 4^f^	36 (82)	28 (64)*	17 (65)	13 (50)
PROMIS T‐score < 40^g^	14 (37)	11 (29)	8 (36)	8 (36)
REBS^a^^,^ h				
Intrinsic	4.75 (4.44–5.00)	5.00 (4.25–5.00)	4.50 (3.13–5.00)	5.00 (3.63–5.00)
Introjected	2.13 (1.00–3.25)	2.00 (1.44–2.63)	2.00 (1.50–2.75)	3.00 (2.00–3.50)
Identified	4.75 (4.00–5.00)	4.50 (4.25–5.00)	4.75 (4.25–5.00)	4.75 (4.38–5.00)
Integrated	4.38 (3.50–5.00)	4.50 (3.88–5.00)	4.25 (2.88–4.88)	4.00 (3.25–4.88)
External	2.00 (1.00–2.81)	1.88 (1.19–3.00)	2.00 (1.13–3.00)	2.50 (1.75–4.25)
Amotivation	1.00 (1.00–1.56)	1.00 (1.00–1.00)	1.00 (1.00–2.00)	1.00 (1.00–2.00)
BREQ‐2^a^^,^ i				
Intrinsic	4.00 (3.25–4.00)	4.00 (3.25–4.00)	3.50 (2.00–4.00)	3.00 (2.13–4.00)
Introjected	1.00 (0.00–2.00)	1.00 (0.00–2.00)	1.00 (0.00–1.84)	1.00 (0.17–1.00)
Identified	4.00 (3.00–4.00)	3.75 (3.00–4.00)	3.25 (2.88–3.88)	3.00 (2.63–3.63)
External	0.00 (0.00–0.50)	0.00 (0.00–0.50)	0.00 (0.00–0.38)	0.00 (0.00–1.13)
Amotivation	0.00 (0.00–0.00)	0.00 (0.00–0.00)	0.00 (0.00–0.63)	0.00 (0.00–0.00)

*Note:* Data are shown as mean ± SD or *n* (%) unless indicated otherwise.

Abbreviations: BMI, body mass index; DLCO, diffusion capacity of the lungs for carbon monoxide; EQ‐5D, EuroQol five dimensions; FEV_1_, forced expiratory volume in 1 s; FFMI, fat‐free mass index; FMI, fat mass index; FSS, Fatigue Severity Scale; FVC, forced vital capacity; HADS, Hospital Anxiety and Depression Scale; PROMIS PF‐8b, Patient‐Reported Outcomes Measurement Information System Physical Function‐8b; REBS, Regulation of Eating Behaviour Scale.

^a^ Median (IQR).

^b^ Measured in 40/20 patients of the intervention/control group.

^c^ Measured in 25/13 patients of the intervention/control group.

^d^ Measured in 38/23 patients of the intervention/control group.

^e^ Measured in 37/21 patients of the intervention/control group.

^f^ Measured in 44/26 patients of the intervention/control group.

^g^ Measured in 38/22 patients of the intervention/control group.

^h^ Measured in 22/14 patients of the intervention/control group.

^i^ Measured in 23/17 patients of the intervention/control group.

* Significantly different from visit 1, *p* < 0.05.

## 4 Discussion

This study investigated the feasibility of a digital personalised CLI on physical activity levels and dietary quality among patients with PCC. We found that the lifestyle intervention was well‐received by patients with PCC regarding compliance, experiences and perceived effectiveness. Particularly, the individual sessions and educative webinars were well‐attended by patients, and positive experiences concerning the functioning of the coaches, the frequency, content and duration of the sessions and the digital delivery mode were reported. Half of the patients mentioned perceived improvements in their physical activity levels and dietary quality throughout the intervention, with the majority also reporting to have maintained their healthy lifestyle changes after the intervention.

Our findings demonstrate high compliance rates for the individual sessions and educative webinars, while compliance was relatively low for the interactive‐group sessions. Accordingly, comparable attendance rates were found within a group‐based pulmonary telerehabilitation programme, consisting of exercise classes and educative sessions [34]. Previous findings also showed that recovery from PCC was often perceived as isolating, largely due to the limited access to healthcare services and lack of available evidence‐based rehabilitation strategies [35]. This context has consequently fostered significant gratitude among patients with PCC for any support provided by coaches or healthcare professionals [35, 36, 37, 38]. These elements alongside patients' positive experiences regarding the functioning of the coaches, particularly their empathetic support, personalised care and well‐organised sessions, as well as patients' strong motivation to adopt healthier lifestyles driven by their desire to recover from PCC, likely contributed to the high compliance in both individual sessions and educative webinars within our study cohort. While previous studies showed great appreciation for group‐based activities among patients with PCC because of their role in facilitating (emotional) peer support, reducing social isolation and promoting a sense of belonging through shared understanding [36, 37], the lower compliance in the interactive‐group sessions within our study could be explained by the negative ambiance and feelings of guilt when feeling better than others, as reported by some patients.

Our findings indicated that half of the patients reported improvements in physical activity levels and dietary quality during the intervention, with the majority also being able to sustain these lifestyle modifications post‐intervention. Perceived facilitators for adopting healthy lifestyle changes included setting, repeating and evaluating goals, support from loved ones and pacing strategies, while perceived barriers consisted of PCC complaints and increased busyness in daily life. In general, time constraints related to work, household duties and family obligations, along with negative attitudes from loved ones, are well‐recognised barriers to adopting a healthy lifestyle [39]. Additionally, patients identified setbacks in disease recovery as the main pitfall leading to relapses into previous lifestyle habits. Given the fluctuating nature of symptoms in PCC, pacing strategies such as symptom monitoring and adjusting lifestyle behaviours, particularly physical activity levels, accordingly, are essential to prevent overexertion and minimise the risk of setbacks in recovery among patients with PCC [40, 41].

Most patients reported a positive experience with the programme, valuing the personalised content related to diet and physical activity as well as the interactive approach of the individual sessions, the opportunity to share experiences with others on topics related to PCC complaints and recovery (i.e., healthy lifestyle tips) during the interactive‐group sessions, and the content of the educative webinars. The monthly individual counselling allowed for the provision of tailored content, thereby addressing patients' specific requirements, which aligns with current guidelines emphasising the need for personalised interventions considering the multifaceted nature of PCC [40]. Although the content of the educative webinars was valued among patients, the sessions could be improved by including content on mental health problems and implementing a greater interactive approach. This is particularly relevant as the detrimental effects of PCC on psychological outcomes such as anxiety, depression, sleep disturbances and post‐traumatic stress disorder are well‐documented [42, 43]. While this study primarily focused on lifestyle, a holistic approach that also integrates support for both physical and psychological symptom management is essential to further enhance overall well‐being among patients with PCC [40, 44]. Furthermore, a greater interactive learning environment could be fostered using social constructivist approaches, facilitating participants' understanding of the content by engaging with personal stories and life experiences [45]. Lastly, the overall duration of the 9‐month intervention, the frequency and duration of the different sessions, as well as the digital communication medium were positively received among most patients. These findings confirm previous research showing the acceptance and feasibility of digital interventions among patients with PCC [36, 46, 47]. The timing and duration of the interactive‐group sessions and educative webinars could, however, be earlier in the day and shortened, as some patients experienced persisting concentration problems and fatigue. Offering these sessions at varying times and durations, tailored to individual recovery stages, may therefore be advisable. This approach may also reduce feelings of negativity and guilt in group sessions, as patients can select sessions that align with their current needs, thereby increasing the sessions' relevance and effectiveness.

Based on the findings of this study and current clinical guidelines, PCC management requires a multifaceted, patient‐tailored approach [40, 44]. CLIs focus on various components tailored to individual needs such as lifestyle (i.e., exercise and nutrition), education (i.e., symptom and sleep management), group support and behaviour change, which has already been shown to positively impact lifestyle changes (e.g., increased physical activity levels and healthy dietary behaviour), BMI/weight and quality of life among various chronic disease conditions [31, 48, 49, 50]. Additionally, current literature has demonstrated the positive impact of digital physical activity counselling during the Covid‐19 pandemic, both in physically inactive adults and specifically in post‐Covid‐19 patients, on physical activity levels and healthy lifestyle behaviours, consistent with our findings [51, 52]. While these studies were also primarily delivered by researchers via digital platforms, they were of relatively shorter duration (up to 6 months) and did not include a dietary component [51, 52], which is a key element of a well‐balanced healthy lifestyle, as compared to our study. Our results further add to the existing literature by showing the feasibility of a digital CLI in patients with PCC, thereby suggesting the potential of implementing CLIs in the management of PCC by improving physical and psychological well‐being, while also promoting long‐term healthy lifestyle behaviour changes.

CLIs may be improved by incorporating tailored nutritional strategies to promote muscle mass recovery and alleviate persistent inflammation following severe SARS‐CoV‐2 infection [11]. This study explored the feasibility of an NS among patients with PCC, demonstrating good compliance in 16 of the 46 patients (35%). Several factors, including dissatisfaction with the taste, the high‐caloric density counteracting weight loss goals and the onset of nausea, contributed to the low compliance rates. As most patients were overweight, the NS, originally developed to combat disease‐related malnutrition [53], might be less feasible within this patient population. Additionally, the natural recovery in FFMI, independent of the NS, suggests that additional tailored nutrition incorporating protein may be less relevant for PCC recovery. Furthermore, improvements in pulmonary function were observed over time in the intervention group with NS. Specific interaction effects could, however, not be established due to the small sample size and associated power constraints, and further research employing a larger randomised study design is warranted to investigate the effects of the NS on health outcomes in patients with PCC.

Our study demonstrates the feasibility of a digital personalised CLI for patients with PCC regarding compliance, experiences and perceived effects on improving physical activity and dietary quality. The 9‐month follow‐up period allowed us to support longer‐term lifestyle (behavioural) modifications in this population. Furthermore, the use of methodological triangulation, incorporating both questionnaires and interviews, provided a robust and reliable representation of the interventions' feasibility. Nevertheless, some limitations of this study deserve discussion. Firstly, our cohort consisted of a selected sample of primarily hospitalised COVID‐19 patients who attended the outpatient clinic and possessed digital literacy skills necessary to participate in the intervention, which limits the generalisability of our findings to the broader population of patients with PCC. Secondly, due to practical issues, physical activity levels could not be assessed, limiting our ability to evaluate participants' objective improvements. Thirdly, variation in survey completion methods (online or on paper) may have influenced results, which should be considered when interpreting the study findings. Additionally, participation in the lifestyle intervention was voluntary, potentially resulting in a cohort comprising the most motivated patients, which may have introduced some selection bias. Some sampling bias may also have occurred in the process evaluation, as patients with particularly positive or negative experiences are more likely to participate in interviews. To mitigate sampling bias, we made use of a balanced interview selection considering gender and timing of completing the intervention. One of the researchers (D.G.) involved in the data analysis and study description also served as a coach during the intervention, which may have introduced reflexivity bias. To preserve the validity of the findings, this researcher (D.G.) refrained from conducting interviews with patients for whom she served as a coach. The variation in time (1–13 months), between completing the intervention and the process evaluation, may have influenced the results and led to some recall bias. Future studies could consider using a homogenised follow‐up period for the process evaluation to prevent recall bias and obtain more comparable results.

## 5 Conclusion

The present study showed that a digital personalised CLI was well‐received by patients with PCC in terms of compliance, experiences and perceived effectiveness. Particularly, the individual sessions and educative webinars were well‐attended by patients, and positive experiences were reported regarding the functioning of the coaches, the various sessions, as well as the digital delivery mode. Half of the patients reported improvements in physical activity levels and dietary quality during the intervention, with the majority also reporting sustainment of these lifestyle changes after the intervention. These findings suggest the integration of digital personalised CLIs into routine post‐Covid‐19 care to enhance patient recovery. Future studies with a larger cohort and randomisation designs are, however, still needed to unveil the effects of such long‐term lifestyle interventions on objective health outcomes among patients with PCC, which may contribute to the development and implementation of personalised interventions in the future.

## Author Contributions

**Debbie Gach:** conceptualisation, investigation, writing – original draft, methodology, data curation, formal analysis, visualisation, writing – review and editing. **Charlotte D. C. Born:** writing – original draft, data curation, investigation, writing – review and editing, methodology, visualisation. **Lisanne L. T. Schuurman:** investigation, data curation, writing – review and editing. **Frits H. M. Osch:** writing – review and editing, visualisation, methodology, supervision. **Joop P. den Bergh:** writing – review and editing, supervision, methodology, visualisation. **Sanne M. P. L. Gerards:** conceptualisation, methodology, visualisation, writing – review and editing, supervision. **Rik Crutzen:** conceptualisation, methodology, visualisation, writing – review and editing, supervision. **Annemie M. W. J. Schols:** conceptualisation, funding acquisition, writing – review and editing, methodology, visualisation, supervision. **Rosanne J. H. C. G. Beijers:** conceptualisation, funding acquisition, investigation, methodology, visualisation, writing – review and editing, supervision, data curation.

## Conflicts of Interest

D. Gach, C. Born, A. Schols and R. Beijers are part of the P4O2 consortium, which is financially supported by a public and private partnership sponsored by Health Holland. J. van den Bergh receives research funding from UCB and Amgen (outside P4O2). No conflicts of interest exist for the other authors.

## Supporting information

P4O2 COVID lifestyle intervention paper supplementary final Health Expectations Final Publication.

Figure S1.

## Data Availability

Datasets and scripts used in this study are available from the corresponding author upon request.

## References

[1] A. V. Ballering , S. K. R. van Zon , T. C. Olde Hartman , and J. G. M. Rosmalen , “Persistence of Somatic Symptoms After COVID‐19 in the Netherlands: An Observational Cohort Study,” Lancet 400, no. 10350 (2022): 452–461. 35934007 10.1016/S0140-6736(22)01214-4 PMC9352274

[2] J. B. Soriano , S. Murthy , J. C. Marshall , P. Relan , and J. V. Diaz , “A Clinical Case Definition of Post‐COVID‐19 Condition by a Delphi Consensus,” Lancet Infectious Diseases 22, no. 4 (2022): e102–e107. 34951953 10.1016/S1473-3099(21)00703-9 PMC8691845

[3] J. Li , Y. Zhou , J. Ma , et al., “The Long‐Term Health Outcomes, Pathophysiological Mechanisms and Multidisciplinary Management of Long COVID,” Signal Transduction and Targeted Therapy 8, no. 1 (2023): 416. 37907497 10.1038/s41392-023-01640-z PMC10618229

[4] E. Wynberg , H. D. G. van Willigen , M. Dijkstra , et al., “Evolution of Coronavirus Disease 2019 (COVID‐19) Symptoms During the First 12 Months After Illness Onset,” Clinical Infectious Diseases 75, no. 1 (2022): e482–e490. 34473245 10.1093/cid/ciab759 PMC8522402

[5] S. Wang , Y. Li , Y. Yue , et al., “Adherence to Healthy Lifestyle Prior to Infection and Risk of Post‐COVID‐19 Condition,” JAMA Internal Medicine 183, no. 3 (2023): 232–241. 36745445 10.1001/jamainternmed.2022.6555 PMC9989904

[6] A. F. Cobre , M. Surek , R. O. Vilhena , et al., “Influence of Foods and Nutrients on COVID‐19 Recovery: A Multivariate Analysis of Data From 170 Countries Using a Generalized Linear Model,” Clinical Nutrition 41, no. 12 (2022): 3077–3084. 33933299 10.1016/j.clnu.2021.03.018 PMC7982641

[7] N. Feter , E. L. Caputo , F. M. Delpino , et al., “Physical Activity and Long Covid: Findings From the Prospective Study About Mental and Physical Health in Adults Cohort,” Public Health 220 (2023): 148–154. 37320945 10.1016/j.puhe.2023.05.011 PMC10263464

[8] H. Humphreys , L. Kilby , N. Kudiersky , and R. Copeland , “Long COVID and the Role of Physical Activity: A Qualitative Study,” BMJ Open 11, no. 3 (2021): e047632. 10.1136/bmjopen-2020-047632 PMC7948149 33692189

[9] J. Wright , S. Astill , and M. Sivan , “The Relationship Between Physical Activity and Long COVID: A Cross‐Sectional Study,” International Journal of Environmental Research and Public Health 19, no. 9 (2022): 5093. 35564488 10.3390/ijerph19095093 PMC9105041

[10] J. T. Mey , J. P. Kirwan , and C. L. Axelrod , “The Role of Nutrition in Mitigating the Effects of COVID‐19 From Infection Through PASC,” Nutrients 15, no. 4 (2023): 866. 36839224 10.3390/nu15040866 PMC9961621

[11] L. Barrea , W. B. Grant , E. Frias‐Toral , et al., “Dietary Recommendations for Post‐COVID‐19 Syndrome,” Nutrients 14, no. 6 (2022): 1305. 35334962 10.3390/nu14061305 PMC8954128

[12] A. J. Shetty , M. Banerjee , T. N. Prasad , S. K. Bhadada , and R. Pal , “Do Vitamin D Levels or Supplementation Play A Role in COVID‐19 Outcomes?—A Narrative Review,” Annals of Palliative Medicine 13, no. 1 (2024): 162–177. 38124476 10.21037/apm-23-113

[13] S. Doaei , A. Mardi , and M. Zare , “Role of Micronutrients in the Modulation of Immune System and Platelet Activating Factor in Patients With COVID‐19; A Narrative Review,” Frontiers in Nutrition 10 (2023): 1207237. 37781112 10.3389/fnut.2023.1207237 PMC10540693

[14] B. M. Peterson , I. Unger , S. Sun , et al., “The Vital Role of Exercise and Nutrition in COVID‐19 Rehabilitation: Synergizing Strength,” Frontiers in Sports and Active Living 5 (2023): 1305175. 38143784 10.3389/fspor.2023.1305175 PMC10748488

[15] M. T. Williams , T. Effing , C. Paquet , et al., “Counseling for Health Behavior Change in People With COPD: Systematic Review,” International Journal of Chronic Obstructive Pulmonary Disease 12 (2017): 2165–2178. 28794621 10.2147/COPD.S111135 PMC5536233

[16] E. L. Deci and R. M. Ryan , “The ‘What’ and ‘Why’ of Goal Pursuits: Human Needs and the Self‐Determination of Behavior,” Psychological Inquiry 11, no. 4 (2000): 227–268.

[17] W. R. Miller and S. Rollnick , Motivational Interviewing: Helping People Change (Guildford Press, 2013).

[18] J. Li , W. Xia , C. Zhan , et al., “A Telerehabilitation Programme in Post‐Discharge COVID‐19 Patients (TERECO): A Randomised Controlled Trial,” Thorax 77, no. 7 (2022): 697–706. 34312316 10.1136/thoraxjnl-2021-217382 PMC8318721

[19] J. L. Smith , K. Deighton , A. Q. Innes , et al., “Improved Clinical Outcomes in Response to a 12‐Week Blended Digital and Community‐Based Long‐COVID‐19 Rehabilitation Programme,” Frontiers in Medicine 10 (2023): 1149922. 37293307 10.3389/fmed.2023.1149922 PMC10244528

[20] J. Calvo‐Paniagua , M. J. Díaz‐Arribas , J. A. Valera‐Calero , et al., “A Tele‐Health Primary Care Rehabilitation Program Improves Self‐Perceived Exertion in COVID‐19 Survivors Experiencing Post‐Covid Fatigue and Dyspnea: A Quasi‐Experimental Study,” PLoS One 17, no. 8 (2022): e0271802. 35926004 10.1371/journal.pone.0271802 PMC9352012

[21] C. Colas , M. Bayle , P. Labeix , et al., “Management of Long COVID—The Covimouv' Pilot Study: Importance of Adapted Physical Activity for Prolonged Symptoms Following SARS‐CoV2 Infection,” Frontiers in Sports and Active Living 4 (2022): 877188. 35847457 10.3389/fspor.2022.877188 PMC9283867

[22] C. Derksen , R. Rinn , L. Gao , et al., “Longitudinal Evaluation of an Integrated Post‐COVID‐19/Long COVID Management Program Consisting of Digital Interventions and Personal Support: Randomized Controlled Trial,” Journal of Medical Internet Research 25 (2023): e49342. 37792437 10.2196/49342 PMC10563866

[23] E. Pehlivan , I. Palalı , S. G. Atan , D. Turan , H. Çınarka , and E. Çetinkaya , “The Effectiveness of Post‐Discharge Telerehabilitation Practices in COVID‐19 Patients: Tele‐COVID Study‐Randomized Controlled Trial,” Annals of Thoracic Medicine 17, no. 2 (2022): 110–117. 35651892 10.4103/atm.atm_543_21 PMC9150661

[24] M. Imamura , A. R. Mirisola , F. Q. Ribeiro , et al., “Rehabilitation of Patients After COVID‐19 Recovery: An Experience at the Physical and Rehabilitation Medicine Institute and Lucy Montoro Rehabilitation Institute,” Clinics 76 (2021): e2804. 34133481 10.6061/clinics/2021/e2804 PMC8183312

[25] E. Daynes , C. Gerlis , E. Chaplin , N. Gardiner , and S. J. Singh , “Early Experiences of Rehabilitation for Individuals Post‐Covid to Improve Fatigue, Breathlessness Exercise Capacity and Cognition—A Cohort Study,” Chronic Respiratory Disease 18 (2021): 14799731211015691. 33957805 10.1177/14799731211015691 PMC8114752

[26] M. Gobbi , E. Bezzoli , F. Ismelli , et al., “Skeletal Muscle Mass, Sarcopenia and Rehabilitation Outcomes in Post‐Acute COVID‐19 Patients,” Journal of Clinical Medicine 10, no. 23 (2021): 5623. 34884325 10.3390/jcm10235623 PMC8658326

[27] M. A. Valverde‐Martinez , R. Lopez‐Liria , J. Martinez‐Cal , M. J. Benzo‐Iglesias , L. Torres‐Alamo , and P. Rocamora‐Perez , “Telerehabilitation, a Viable Option in Patients With Persistent Post‐COVID Syndrome: A Systematic Review,” Healthcare 11, no. 2 (2023): 187. 36673555 10.3390/healthcare11020187 PMC9859291

[28] N. Baalbaki , J. M. Blankestijn , M. I. Abdel‐Aziz , et al., “Precision Medicine for More Oxygen (P4O2)‐Study Design and First Results of the Long COVID‐19 Extension,” Journal of Personalized Medicine 13, no. 7 (2023): 1060. 37511673 10.3390/jpm13071060 PMC10381397

[29] M. van Beers , M. P. M. H. Rutten‐Van mölken , C. van de Bool , et al., “Clinical Outcome and Cost‐Effectiveness of a 1‐Year Nutritional Intervention Programme in COPD Patients With Low Muscle Mass: The Randomized Controlled Nutrain Trial,” Clinical Nutrition 39, no. 2 (2020): 405–413. 30954363 10.1016/j.clnu.2019.03.001

[30] C. E. van Rinsum , S. M. P. L. Gerards , G. M. Rutten , I. A. M. van de Goor , and S. P. J. Kremers , “The Coaching on Lifestyle (CooL) Intervention for Obesity, a Study Protocol for an Action‐Oriented Mixed‐Methods Study,” BMC Public Health 18, no. 1 (2018): 117. 29310640 10.1186/s12889-017-5010-4 PMC5759228

[31] C. van Rinsum , S. Gerards , G. Rutten , et al., “The Coaching on Lifestyle (CooL) Intervention for Overweight and Obesity: A Longitudinal Study Into Participants' Lifestyle Changes,” International Journal of Environmental Research and Public Health 15, no. 4 (2018): 680. 29617337 10.3390/ijerph15040680 PMC5923722

[32] T. Cederholm , G. L. Jensen , M. I. T. D. Correia , et al., “GLIM Criteria for the Diagnosis of Malnutrition—A Consensus Report From the Global Clinical Nutrition Community,” Clinical Nutrition 38, no. 1 (2019): 1–9. 30181091 10.1016/j.clnu.2018.08.002

[33] A. Giorgi , The Descriptive Phenomenological Method in Psychology (Duquesne University Press, 2009).

[34] A. J. Simpson , A. Green , M. Nettleton , et al., “Group‐Based Pulmonary Telerehabilitation Is Feasible, Safe, Beneficial and Well‐Received in Patients Who Have Been Hospitalised With COVID‐19,” ERJ Open Research 9, no. 2 (2023): 00373‐2022. 36915803 10.1183/23120541.00373-2022 PMC9703872

[35] E. Duncan , L. Alexander , J. Cowie , et al., “Investigating Scottish Long COVID Community Rehabilitation Service Models From the Perspectives of People Living With Long COVID and Healthcare Professionals: A Qualitative Descriptive Study,” BMJ Open 13, no. 12 (2023): e078740. 10.1136/bmjopen-2023-078740 PMC10729197 38101833

[36] C. Killingback , M. Thompson , M. Nettleton , et al., “Telerehabilitation for Patients Who Have Been Hospitalised With Covid‐19: A Qualitative Study,” Disability and Rehabilitation 46, no. 1 (2024): 150–158. 36629074 10.1080/09638288.2022.2159075

[37] C. Gerlis , A. Barradell , N. Y. Gardiner , et al., “The Recovery Journey and the Rehabilitation Boat—A Qualitative Study to Explore Experiences of COVID‐19 Rehabilitation,” Chronic Respiratory Disease 19 (2022): 14799731221114266. 35850558 10.1177/14799731221114266 PMC9297070

[38] H. Al‐Jabr , D. R. Thompson , D. J. Castle , and C. F. Ski , “Experiences of People With Long COVID: Symptoms, Support Strategies and the Long COVID Optimal Health Programme (LC‐OHP),” Health Expectations 27, no. 1 (2024): e13879. 37751413 10.1111/hex.13879 PMC10726154

[39] M. Subramaniam , F. Devi , P. V. AshaRani , et al., “Barriers and Facilitators for Adopting a Healthy Lifestyle in a Multi‐Ethnic Population: A Qualitative Study,” PLoS One 17, no. 11 (2022): e0277106. 36322596 10.1371/journal.pone.0277106 PMC9629631

[40] N. England . Commissioning Guidance for Post‐COVID Services for Adults, Children and Young People 2024, https://www.england.nhs.uk/long-read/commissioning-guidance-for-post-covid-services-for-adults-children-and-young-people/#principles-of-care-for-long-covid.

[41] J. DeMars , D. A. Brown , I. Angelidis , et al., “What Is Safe Long COVID Rehabilitation?,” Journal of Occupational Rehabilitation 33, no. 2 (2023): 227–230. 36315323 10.1007/s10926-022-10075-2 PMC9628454

[42] M. Taquet , Z. Skorniewska , T. De Deyn , et al, “Cognitive and Psychiatric Symptom Trajectories 2‐3 Years After Hospital Admission for COVID‐19: A Longitudinal, Prospective Cohort Study in the UK,” Lancet Psychiatry 11, no. 9 (2024): 696–708. 39096931 10.1016/S2215-0366(24)00214-1 PMC7618856

[43] M. Marchi , P. Grenzi , V. Serafini , et al., “Psychiatric Symptoms in Long‐Covid Patients: A Systematic Review,” Frontiers in Psychiatry 14 (2023): 1138389. 37415689 10.3389/fpsyt.2023.1138389 PMC10320160

[44] WHO , Clinical Management of COVID‐19 (Living Guideline, 2023). 35917394

[45] M. Saunders , Learning Theories: Understanding How People Learn (Windsor & Down Press, 2020).

[46] P. H. I. Lloyd‐Evans , M. M. Baldwin , E. Daynes , et al, “Early Experiences of the Your COVID Recovery((R)) Digital Programme for Individuals With Long COVID,” BMJ Open Respiratory Research 9, no. 1 (2022): e001237. 10.1136/bmjresp-2022-001237 PMC9527747 36171050

[47] M. J. Estebanez‐Perez , R. Martin‐Valero , P. Pastora‐Estebanez , and J. M. Pastora‐Bernal , “Experiences of People With Long Covid With a Digital Physiotherapy Intervention: A Qualitative Study,” Health Expectations 27, no. 2 (2024): e13993. 38590093 10.1111/hex.13993 PMC11002316

[48] C. D. C. Born , R. Bhadra , G. D'Souza , et al., “Combined Lifestyle Interventions in the Prevention and Management of Asthma and COPD: A Systematic Review,” Nutrients 16, no. 10 (2024): 1515. 38794757 10.3390/nu16101515 PMC11124109

[49] Y. Hassan , V. Head , D. Jacob , M. O. Bachmann , S. Diu , and J. Ford , “Lifestyle Interventions for Weight Loss in Adults With Severe Obesity: A Systematic Review,” Clinical Obesity 6, no. 6 (2016): 395–403. 27788558 10.1111/cob.12161

[50] N. Lv , K. M. J. Azar , L. G. Rosas , S. Wulfovich , L. Xiao , and J. Ma , “Behavioral Lifestyle Interventions for Moderate and Severe Obesity: A Systematic Review,” Preventive Medicine 100 (2017): 180–193. 28450123 10.1016/j.ypmed.2017.04.022 PMC5503454

[51] L. Goncalves , M. S. Moraes , and D. A. S. Silva , “Counseling for Physical Activity in Adults During the COVID‐19 Pandemic: A Scope Review,” International Journal of Environmental Research and Public Health 19, no. 14 (2022): 05003. 10.3390/ijerph19148687 PMC9322393 35886538

[52] C. Y. Lai , C. H. Lin , T. C. Chao , et al., “Effectiveness of a 12‐Week Telerehabilitation Training in People With Long COVID: A Randomized Controlled Trial,” Annals of Physical and Rehabilitation Medicine 67, no. 5 (2024): 101853. 38824899 10.1016/j.rehab.2024.101853

[53] A. Laviano , P. C. Calder , A. M. W. J. Schols , F. Lonnqvist , M. Bech , and M. Muscaritoli , “Safety and Tolerability of Targeted Medical Nutrition for Cachexia in Non‐Small‐Cell Lung Cancer: A Randomized, Double‐Blind, Controlled Pilot Trial,” Nutrition and Cancer 72, no. 3 (2020): 439–450. 31290697 10.1080/01635581.2019.1634746

