# Supplementary material for: A Mixed‐Methods Study Exploring the Feasibility of a Digital Combined Lifestyle Intervention for Patients With Post Covid‐19 Condition
Source: Health Expect. 2025 May 25;28(3):e70299. doi: 10.1111/hex.70299 (PMC12104566; doi:10.1111/hex.70299)
Supplement: Supplementary file 1 — P4O2 COVID lifestyle intervention paper supplementary final Health Expectations Final Publication. [file HEX-28-e70299-s002.docx]

**Supporting information**

**Supporting Information 1**

*Data collection*

1. Health outcomes study visits
   1. Baseline and hospitalization characteristics

During the first study visit at ±3-6 months post COVID-19 infection, general baseline characteristics such as sex, age, ethnicity, smoking status, and presence of co-morbidities were collected via questionnaires and afterwards the gathered information was checked by the researchers using the electronic medical records of the patients. Hospitalization characteristics such as type of admitted ward (ICU or nursing ward), and treatments (oxygen therapy, non-invasive and/or invasive ventilation) received during hospitalization were also retrieved from medical records.

- 1. Body composition

Body composition was assessed during the follow-up study visits at ±3-6 and ±12-15 months post COVID-19 infection. More specifically, anthropometry was assessed by measuring body height and weight (Seca 220, Seca GmbH & Co. KG, Hamburg, Germany) and body mass index (BMI) was subsequently calculated as body weight divided by height squared. Body composition was measured using bioelectrical impedance analysis (BIA; Bodystat 500, EuroMix, Leuven, Belgium). Fat mass (FM) was calculated as total mass subtracted by fat-free mass (FFM). Fat free mass index (FFMI) was calculated as FFM/height squared, and fat mass index (FMI) by FM/height squared.

- 1. Blood samples

Blood samples were also collected during both study visits and stored at minus 80°C for later analyses, which included assessment of fatty acid profile in total phospholipids. The fatty acids contained in the serum samples were converted into their corresponding methyl esters (FAME) by using 3 m HCl in methanol at 85°C for 45 min. After hexane extraction, the fatty acid methyl esters were dissolved in n-hexane and analyzed via gas chromatography using a capillary column (50 m, 0.25 mm, CP7419). Peaks were identified using commercial reference standards.

- 1. Questionnaires

Several questionnaires were administered and completed by the patients during both study visits. If patients were unable to complete the questionnaires during the study visit, the researcher provided a digital link via email, allowing patients to complete the questionnaires at home following the visit. The experienced health status of the patients was measured by the five-level EuroQol five-dimensions questionnaire (EQ-5D-5L). This questionnaire consists of five health domains (mobility, self-care, usual activities, pain or discomfort, and anxiety or depression), each on a five-level scale (no problems, slight problems, moderate problems, severe problems, and extreme problems) [1]. The Dutch value set was used for conversion of EQ-5D scores into utilities [2]. The Hospital Anxiety and Depression Scale (HADS) was used to determine levels of anxiety and depression. A cut-off score >10 on each HADS subscale indicated abnormal levels of anxiety and depression [3,4]. The Fatigue Severity Scale (FSS) was used to assess the severity of fatigue, in which a score ≥4 indicated moderate to high fatigue [5]. Physical functioning was assessed by the Patient-Reported Outcomes Measurement Information System Physical Function-8b (PROMIS PF-8b) questionnaire and a cut-off T-score <40 was considered as low physical functioning [6]. Motivation towards exercising and healthy eating was measured using the Behavioural Regulation in Exercise Questionnaire-2 (BREQ-2) and the Regulation of Eating Behaviour Scale (REBS) [7,8]. Both questionnaires are based on the Self-determination theory (SDT) and answers were given on a 5-point Likert scale [9]. Eventually, five subscale scores related to intrinsic-, introjected-, identified-, integrated-, and external motivation were retrieved for the REBS, and six subscale scores were provided regarding intrinsic-, integrated-, identified-, introjected-, external-, and a-Motivation for the BREQ-2.

- 1. Pulmonary function

Pulmonary function tests were performed during the COVID-19 outpatient clinic visits which took place in parallel to the two follow-up study visits. These tests were performed according to ERS guidelines and compromised pre-bronchodilator spirometry to assess forced expiratory volume in one second (FEV_1_) and forced vital capacity (FVC), as well as the single-breath method to measure diffusion capacity of the lungs for carbon monoxide (DLCO) [10].

*Data processing and analysis*

To evaluate changes in health outcomes between study visit 1 and 2 in the intervention group stratified by NS as well as the control group, a paired samples T-test or Wilcoxon signed rank test was calculated as appropriate for continuous variables, and a McNemar test for ordinal/categorical variables. A one-way ANOVA was used to compare patient characteristics of the intervention group stratified by process evaluation and in case of a significant group effect, pairwise comparison was done using a post hoc Bonferroni test.

**Supporting Information 2**

**
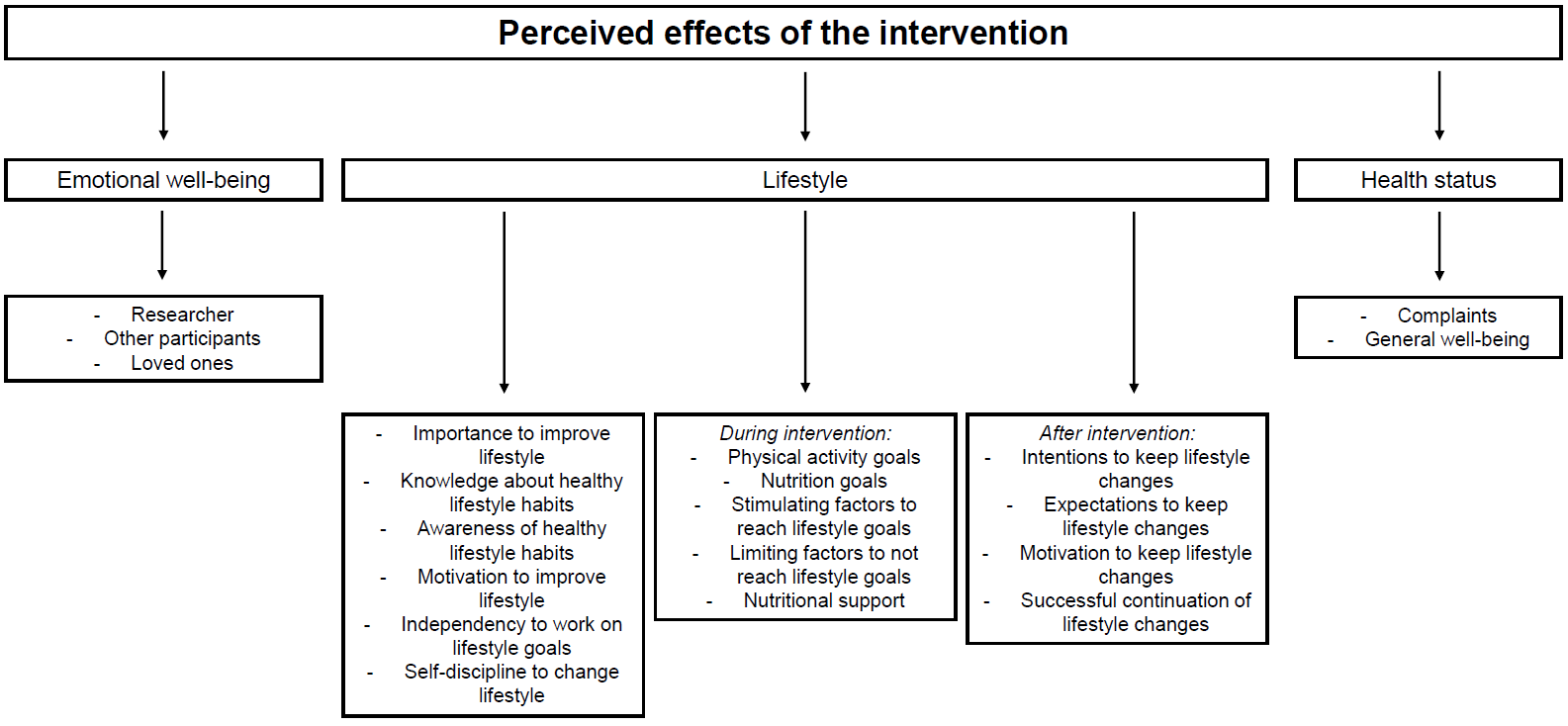

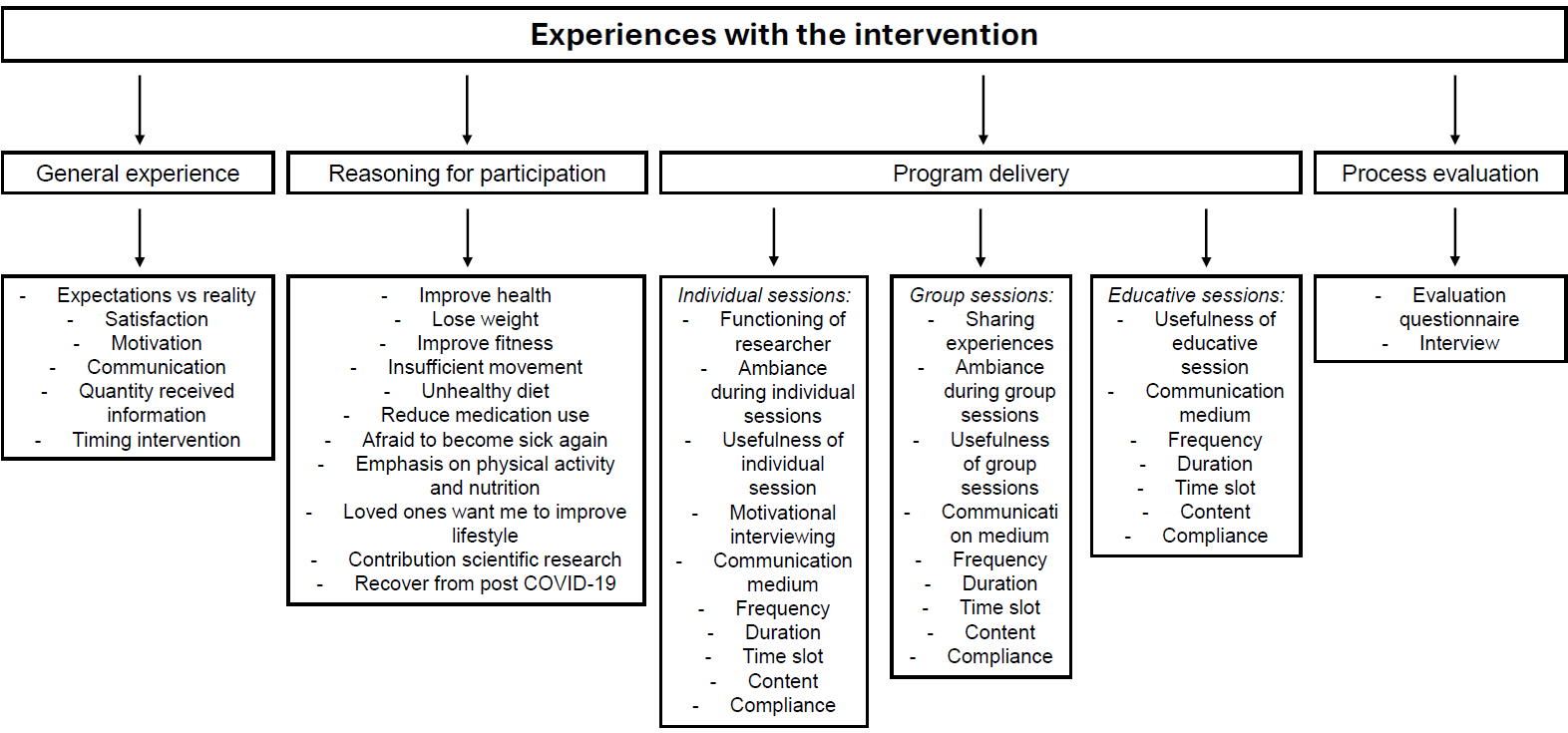
Figure S1. Coding tree**

**Supporting Information 3**

| **Health outcomes**  **study visits** |  | |  |  | |  |  |  |  |
| --- | --- | --- | --- | --- | --- | --- | --- | --- | --- |
|  | ***Intervention with NS***  ***(n=16)*** | |  | ***Intervention without NS***  ***(n=28)*** | |  | ***Control***  ***(n=28)*** | |  |
|  | **Visit 1** | **Visit 2** | ***p*-value** | **Visit 1** | **Visit 2** | ***p*-value** | **Visit 1** | **Visit 2** | ***p*-value** |
| **Body composition^b^** |  |  |  |  |  |  |  |  |  |
| BMI in kg/m^2^ | 31.1 (26.9 – 31.9)^a^ | 30.9 (27.1 – 33.7)^a^ | 0.064 | 29.8±5.1 | 30.1±4.9 | 0.475 | 30.0±4.4 | 30.4±4.4 | 0.192 |
| FFMI in kg/m^2^ | 20.0±2.3 | 20.4±2.4 | **0.038** | 19.2±2.7 | 19.8±2.7 | **0.013** | 19.7±1.9 | 20.4±2.1 | **0.026** |
| FMI in kg/m^2^ | 9.8 (8.0 – 13.6)^a^ | 10.2 (7.9 – 13.5)^a^ | 0.507 | 10.6±3.3 | 10.2±3.4 | 0.136 | 10.3±3.7 | 9.9±3.6 | 0.279 |
| **Pulmonary function^c^** |  |  |  |  |  |  |  |  |  |
| FEV_1_ in %pred | 78.5±20.0 | 84.7±19.5 | **0.015** | 88.9±18.3 | 92.0±10.5 | 0.304 | 83.0±13.8 | 85.7±14.0 | 0.214 |
| FVC in %pred | 81.0±23.9 | 88.2±21.1 | **0.006** | 86.2±21.4 | 89.6±14.0 | 0.268 | 82.1±13.7 | 84.7±11.3 | 0.222 |
| FEV_1_/FVC in % | 97.4±16.5 | 101.5±27.2 | 0.448 | 99.0±16.7 | 102.8±10.1 | 0.394 | 100.4±13.5 | 99.5±13.2 | 0.588 |
| DLCO in %pred | 70.4±15.9 | 79.3±15.1 | **0.003** | 78.5±23.8 | 84.2±15.6 | 0.141 | 65.8±16.9 | 66.5±16.8 | 0.174 |
| **Questionnaires** |  |  |  |  |  |  |  |  |  |
| EQ-5D score^da^ | 0.80 (0.62 – 0.90) | 0.89 (0.75 – 0.91) | 0.202 | 0.74 (0.63 – 0.89) | 0.85 (0.71 – 1.00) | 0.131 | 0.85 (0.68 – 1.00) | 0.89 (0.68 – 1.00) | 0.691 |
| FSS ≥4^e^ | 14 (93) | 10 (67) | 0.125 | 19 (73) | 15 (58) | 0.289 | 17 (65) | 13 (50) | 0.219 |

**Table S1: Health outcomes of the intervention group stratified by NS and control group at study visit 1 and 2.**

**Note:** Data are shown as mean±SD or n (%) unless indicated otherwise. ^a^Median (IQR). ^b^Measured in 13/26/20 patients of the intervention with NS/Intervention without NS/control group. ^c^Measured in 12/11/13 patients of the intervention with NS/Intervention without NS/control group. ^d^Measured in 12/23/23 patients of the intervention with NS/Intervention without NS/control group. ^e^Measured in 15/26/26 patients of intervention with NS/Intervention without NS/control group. Bold indicates a significant difference between visit 1 and 2, *p*<0.05. Abbreviations: BMI, body mass index; DLCO, diffusion capacity of the lungs for carbon monoxide; EQ-5D, EuroQol five-dimensions; FEV_1_, forced expiratory volume in one second; FFMI, fat free mass index; FMI, fat mass index; FSS, Fatigue Severity Scale; FVC, forced vital capacity; NS, nutritional supplement.

**Supporting Information 4**

*Patient characteristics process evaluation*

Less white patients were present in the intervention without evaluation group as compared to the group with evaluationQ, as well as the group with evaluationQ and interview (*p*=0.007 and *p*=0.022, respectively; Table S2). More ex-smokers were present in patients who completed the evaluationQ and interview as compared to patients who did not perform the evaluation (*p*=0.011).

**Table S2: Patient characteristics of the intervention group stratified by process**

**evaluation.**

| **Patient characteristics** | **Intervention with**  **evaluationQ**  **(n=20)** | **Intervention with evaluationQ and interview**  **(n=13)** | **Intervention without evaluation (n=14)** | ***p*-value** |
| --- | --- | --- | --- | --- |
| Age in years | 55.1±5.5 | 56.4±6.0 | 52.5±6.3 | 0.918 |
| Male | 8 (40) | 4 (31) | 7 (50) | 0.595 |
| Ethnicity^a^ |  |  |  | **0.005** |
| *White* | 18 (95)* | 12 (92)* | 7 (54) |  |
| *Other* | 1 (5) | 1 (8) | 6 (46) |  |
| Smoking status |  |  |  | **0.012** |
| *Current* | 0 (0) | 0 (0) | 0 (0) |  |
| *Ex* | 11 (55) | 10 (77)* | 3 (21) |  |
| *Never* | 9 (45) | 3 (23) | 11 (79) |  |
| BMI in kg/m^2^ | 30.0±5.1 | 30.4±3.7 | 31.1±8.2 | 0.103 |
| BMI categories |  |  |  | 0.475 |
| *Normal weight (18.5-25.0 kg/m²)* | 2 (10) | 0 (0) | 3 (21) |  |
| *Overweight (25.0-30.0 kg/m²)* | 8 (40) | 6 (46) | 4 (29) |  |
| *Obese (≥ 30.0 kg/m²)* | 10 (50) | 7 (54) | 7 (50) |  |

**Note:** Data are shown as mean±SD or n (%). ^a^Assessed in 19/13/13 of the intervention with evaluationQ/with evaluationQ and interview/without evaluation. *Significantly different from intervention without evaluation, *p*<0.05. Abbreviations: BMI, body mass index.

**Supporting Information 5**

**Table S3: Results of the evaluationQ**

| **Reasons participation in lifestyle intervention** | | **n=33** |
| --- | --- | --- |
| 1. I want to become healthier | | 16/33 (49) |
| 1. I want to lose weight | | 9/33 (27) |
| 1. I want to improve my physical conditioning | | 16/33 (49) |
| 1. I want to increase my physical activity level | | 4/33 (12) |
| 1. I want to eat healthier | | 2/33 (6) |
| 1. I want to get insights into a healthier lifestyle | | 4/33 (12) |
| 1. I want to use less/no medication | | 4/33 (12) |
| 1. I am afraid to become sick again | | 8/33 (24) |
| 1. I want to recover from post COVID-19 condition | | 6/33 (18) |
| 1. I want to contribute to scientific research | | 2/33 (6) |
| 1. Because of the combined focused on improving diet and physical activity | | 12/33 (36) |
| 1. Because my loved ones want me to improve my lifestyle | | 0/33 (0) |
| **Part A. General statements about the experiences with the lifestyle intervention** | |  |
| 1. I had … information to make a good decision about participating in the lifestyle intervention | |  |
| - *Sufficient* | | 33/33 (100) |
| - *Insufficient* | | 0/0 (0) |
| 1. The lifestyle intervention … based on the obtained information | |  |
| - *Did go as expected* | | 32/33 (97) |
| - *Did not go as expected* | | 1/33 (3) |
| 1. I am … about the course of the lifestyle intervention | |  |
| - *Satisfied* | | 31/32 (94) |
| - *Not satisfied* | | 1/32 (3) |
| 1. I am … about the communication with the coaches during the lifestyle intervention | |  |
| - *Satisfied* | | 33/33 (100) |
| - *Not satisfied* | | 0/0 (0) |
| 1. I am … about the functioning of the coaches during the lifestyle intervention | |  |
| - *Satisfied* | | 33/33 (100) |
| - *Not satisfied* | | 0/0 (0) |
| **Part B. General statements about lifestyle** |  |  |
| 1. Before the start of the intervention, I was … to work on my lifestyle |  |  |
| - *Motivated* | 32/33 (97) |  |
| - *Not motivated* | 1/33 (3) |  |
| 1. During the intervention, I was … to work on my lifestyle |  |  |
| - *Motivated* | 31/33 (94) |  |
| - *Not motivated* | 2/33 (6) |  |
| 1. I got … |  |  |
| - *Motivated because of the lifestyle intervention* | 23/33 (70) |  |
| - *Not motivated because of the lifestyle intervention* | 0/32 (0) |  |
| - *Motivated because of something else* | 10/33 (30) |  |
| 1. I find it … to work on my lifestyle |  |  |
| - *Important* | 33/33 (100) |  |
| - *Not important* | 0/0 (0) |  |
| 1. I found it … to fulfil the arranged counselling appointments |  |  |
| - *Important* | 33/33 (100) |  |
| - *Not important* | 0/0 (0) |  |
| 1. I find that my lifestyle … during the intervention |  |  |
| - *Improved* | 21/33 (64) |  |
| - *Did not change* | 11/33 (33) |  |
| - *Worsened* | 1/33 (3) |  |
| 1. I am … of the advantages of a healthy lifestyle because of the intervention |  |  |
| - *More aware* | 30/31 (97) |  |
| - *Less aware* | 1/31 (3) |  |
| 1. I got … to work on my lifestyle because of the intervention |  |  |
| - *More motivated* | 30/31 (97) |  |
| - *Less motivated* | 1/31 (3) |  |
| **Part C. Specific statements about physical activity goals** |  |  |
| 1. Before the intervention, I was physically active … |  |  |
| - *Enough* | 20/33 (61) |  |
| - *Not enough* | 13/33 (39) |  |
| 1. During the intervention, I … my physical activity levels |  |  |
| - *Increased* | 17/33 (52) |  |
| - *Remained* | 12/33 (36) |  |
| - *Decreased* | 4/33 (12) |  |
| 1. Because of the intervention, I … my physical activity levels |  |  |
| - *Increased* | 18/29 (62) |  |
| - *Decreased* | 1/29 (3) |  |
| Because of something else than the intervention, I … my physical activity levels |  |  |
| - *Increased* | 6/29 (21) |  |
| - *Decreased* | 4/29 (14) |  |
| 1. I formulated my physical activity goals … |  |  |
| - *Independently* | 24/33 (73) |  |
| - *Not independently* | 9/33 (27) |  |
| 1. I … reach my formulated physical activity goals … |  |  |
| - *Did, Independently* | 19/33 (58) |  |
| - *Did, With help from the coaches* | 8/33 (24) |  |
| - *Did not, -* | 6/33 (18) |  |
| **Part D. Specific statements about dietary intake goals** |  |  |
| 1. Before the intervention, I ate … |  |  |
| - *Healthy* | 24/33 (73) |  |
| - *Not healthy* | 9/33 (27) |  |
| 1. During the intervention, I ate … |  |  |
| - *Healthier* | 18/33 (55) |  |
| - *The same* | 15/33 (45) |  |
| - *Unhealthier* | 0/33 (0) |  |
| 1. Because of the intervention, I ate … |  |  |
| - *Healthier* | 19/27 (70) |  |
| - *Unhealthier* | 0/27 (0) |  |
| Because of something else than the intervention, I ate … |  |  |
| - *Healthier* | 8/27 (30) |  |
| - *Unhealthier* | 0/27 (0) |  |
| 1. I formulated my dietary intake goals … |  |  |
| - *Independently* | 22/33 (67) |  |
| - *Not independently* | 11/33 (33) |  |
| 1. I … reach my formulated dietary intake goals … |  |  |
| - *Did, Independently* | 18/33 (55) |  |
| - *Did, With help from the coaches* | 9/33 (27) |  |
| - *Did not* | 6/33 (18) |  |
| **Part E. Specific statements about the individual counselling sessions** |  |  |
| 1. I found the individual counselling sessions … |  |  |
| - *Useful* | 32/33 (97) |  |
| - *Not useful* | 1/33 (3) |  |
| 1. I found the ambiance during the individual counselling sessions … |  |  |
| - *Pleasant* | 33/33 (100) |  |
| - *Not pleasant* | 0/33 (0) |  |
| 1. The support of the coach during the individual counselling sessions … |  |  |
| - *Has done me a lot of good* | 27/33 (82) |  |
| - *Has not done me a lot good* | 6/33 (18) |  |
| 1. I found it … to have a sympathetic ear during the individual counselling sessions |  |  |
| - *Pleasant* | 32/32 (100) |  |
| - *Not pleasant* | 0/32 (0) |  |
| 1. Contact with the coach … me through the post-COVID-19 period |  |  |
| - *Supported* | 24/33 (73) |  |
| - *Did not support* | 9/33 (27) |  |
| **Part F. Specific statements about the educative webinars** |  |  |
| 1. I found the first educative webinar … |  |  |
| - *Informative and useful* | 20/22 (91) |  |
| - *Not informative and useful* | 2/22 (9) |  |
| 1. A lot of the information given during the first educative webinar was … for me |  |  |
| - *Known* | 15/24 (63) |  |
| - *Not known* | 9/24 (37) |  |
| 1. I found the duration of the first educative webinar … |  |  |
| - *Too long* | 0/23 (0.0) |  |
| - *Too short* | 1/23 (4) |  |
| - *Good* | 22/23 (96) |  |
| 1. I found that the answers to the questions were … during the first educative webinar |  |  |
| - *Good* | 22/22 (100) |  |
| - *Not good* | 0/22 (0) |  |
| 1. I found the second educative webinar … |  |  |
| - *Informative and useful* | 20/23 (87) |  |
| - *Not informative and useful* | 3/23 (13) |  |
| 1. A lot of the information given during the second educative webinar was … for me |  |  |
| - *Known* | 17/23 (74) |  |
| - *Not known* | 6/23 (26) |  |
| 1. I found the duration of the second educative webinar … |  |  |
| - *Too long* | 2/21 (9) |  |
| - *Too short* | 0/21 (0.0) |  |
| - *Good* | 19/21 (91) |  |
| 1. I found that the answers to the questions were … during the second educative webinar |  |  |
| - *Good* | 17/18 (94) |  |
| - *Not good* | 1/18 (6) |  |
| 1. I found the third educative webinar … |  |  |
| - *Informative and useful* | 17/17 (100) |  |
| - *Not informative and useful* | 0/17 (0) |  |
| 1. A lot of the information given during the third educative webinar was … for me |  |  |
| - *Known* | 16/17 (94) |  |
| - *Not known* | 1/17 (6) |  |
| 1. I found the duration of the third educative webinar … |  |  |
| - *Too long* | 0/18 (0) |  |
| - *Too short* | 0/18 (0) |  |
| - *Good* | 18/18 (100) |  |
| 1. I found that the answers to the questions were … during the third educative webinar |  |  |
| - *Good* | 16/16 (100) |  |
| - *Not good* | 0/16 (0) |  |
| 1. I found it … that the educative webinars were digitally |  |  |
| - *Pleasant* | 22/22 (100) |  |
| - *Not pleasant* | 0/22 (0) |  |
| 1. I had … with the digital connection during the educative webinars |  |  |
| - *Problems* | 6/27 (22) |  |
| - *No problems* | 21/27 (78) |  |
| 1. I would have liked … educative webinars during the intervention |  |  |
| - *More* | 2/23 (9) |  |
| - *Less* | 2/23 (9) |  |
| - *The same amount of* | 19/23 (82) |  |
| **Part G. Specific statements about the interactive-group sessions** |  |  |
| 1. I found the interactive-group sessions … |  |  |
| - *Useful* | 16/18 (89) |  |
| - *Not useful* | 2/18 (11) |  |
| 1. I found it … to exchange experiences with other patients in the intervention during the interactive-group sessions |  |  |
| - *Pleasant* | 16/17 (63) |  |
| - *Not pleasant* | 1/17 (37) |  |
| 1. I found the ambiance during the interactive-group sessions … |  |  |
| - *Pleasant* | 18/18 (100) |  |
| - *Not pleasant* | 0/18 (0) |  |
| 1. I found it … that the interactive-group sessions were digitally |  |  |
| - *Pleasant* | 18/18 (100) |  |
| - *Not pleasant* | 0/18 (0) |  |
| 1. I felt … during the interactive-group sessions |  |  |
| - *Safe and secure* | 19/20 (95) |  |
| - *Not safe and secure* | 1/20 (5) |  |
| 1. I had … with the digital connection during the interactive-group sessions |  |  |
| - *Problems* | 1/18 (6) |  |
| - *No problems* | 17/18 (94) |  |
| 1. I found the duration of the interactive-group sessions … |  |  |
| - *Too long* | 2/19 (11) |  |
| - *Too short* | 1/19 (5) |  |
| - *Good* | 16/19 (84) |  |
| 1. I would have liked … interactive-group sessions during the intervention |  |  |
| - *More* | 3/23 (13) |  |
| - *Less* | 2/23 (9) |  |
| - *The same amount of* | 18/23 (78) |  |
| **Part H. Specific statements about continuation of lifestyle changes** |  |  |
| 1. I … to continue with the changes in my movement pattern |  |  |
| - *Intend* | 29/33 (88) |  |
| - *Do not intend* | 1/33 (3) |  |
| - *No changes* | 3/33 (9) |  |
| 1. I think I will … to continue with the changes in my movement pattern independently |  |  |
| - *Succeed* | 27/32 (85) |  |
| - *Not succeed* | 3/32 (9) |  |
| - *No changes* | 2/32 (6) |  |
| 1. I … to continue with the changes in my dietary pattern |  |  |
| - *Intend* | 27/33 (82) |  |
| - *Do not intend* | 2/33 (6) |  |
| - *No changes* | 4/33 (12) |  |
| 1. I think I will … to continue with the changes in my dietary pattern independently |  |  |
| - *Succeed* | 26/32 (81) |  |
| - *Not succeed* | 1/32 (3) |  |
| - *No changes* | 5/32 (16) |  |

**Note:** Data are shown as n (%).

**References**

1. Herdman M, Gudex C, Lloyd A, et al. Development and preliminary testing of the new five-level version of EQ-5D (EQ-5D-5L). *Qual Life Res*. Dec 2011;20(10):1727-36. doi:10.1007/s11136-011-9903-x

2. M MV, K MV, S MAAE, de Wit GA, Prenger R, E AS. Dutch Tariff for the Five-Level Version of EQ-5D. *Value Health*. Jun 2016;19(4):343-52. doi:10.1016/j.jval.2016.01.003

3. Zigmond AS, Snaith RP. The hospital anxiety and depression scale. *Acta Psychiatr Scand*. Jun 1983;67(6):361-70. doi:10.1111/j.1600-0447.1983.tb09716.x

4. Herrmann C. International experiences with the Hospital Anxiety and Depression Scale--a review of validation data and clinical results. *J Psychosom Res*. Jan 1997;42(1):17-41. doi:10.1016/s0022-3999(96)00216-4

5. Krupp LB, LaRocca NG, Muir-Nash J, Steinberg AD. The fatigue severity scale. Application to patients with multiple sclerosis and systemic lupus erythematosus. *Arch Neurol*. Oct 1989;46(10):1121-3. doi:10.1001/archneur.1989.00520460115022

6. PROMIS. Physical Function Scoring Manual. <https://www.healthmeasures.net/images/PROMIS/manuals/Scoring_Manuals_/PROMIS_Physical_Function_Scoring_Manual.pdf>. Updated April 5, 2021. Accessed December 4, 2024.

7. Pelletier LG, Dion SC, Slovinec-D'Angelo M, Reid R. Why do you regulate what you eat? Relationships between forms of regulation, eating behaviors, sustained dietary behavior change, and psychological adjustment. *Motiv Emotion*. Sep 2004;28(3):245-277. doi:Doi 10.1023/B:Moem.0000040154.40922.14

8. Markland D, Tobin V. A modification to the behavioural regulation in exercise questionnaire to include an assessment of amotivation. *J Sport Exercise Psy*. Jun 2004;26(2):191-196. doi:DOI 10.1123/jsep.26.2.191

9. E.L. Deci RMR. *Intrinsic motivation and self-determination in human behavior*. Plenum Press; 1985.

10. Stanojevic S, Kaminsky DA, Miller MR, et al. ERS/ATS technical standard on interpretive strategies for routine lung function tests. *Eur Respir J*. Jul 2022;60(1)doi:10.1183/13993003.01499-2021
