# Supplementary material for: A Mixed‐Methods Study Exploring the Feasibility of a Digital Combined Lifestyle Intervention for Patients With Post Covid‐19 Condition
Source: Health Expect. 2025 May 25;28(3):e70299. doi: 10.1111/hex.70299 (PMC12104566; doi:10.1111/hex.70299)
Supplement: Supplementary file 2 — Figure S1. [file HEX-28-e70299-s001.pdf]

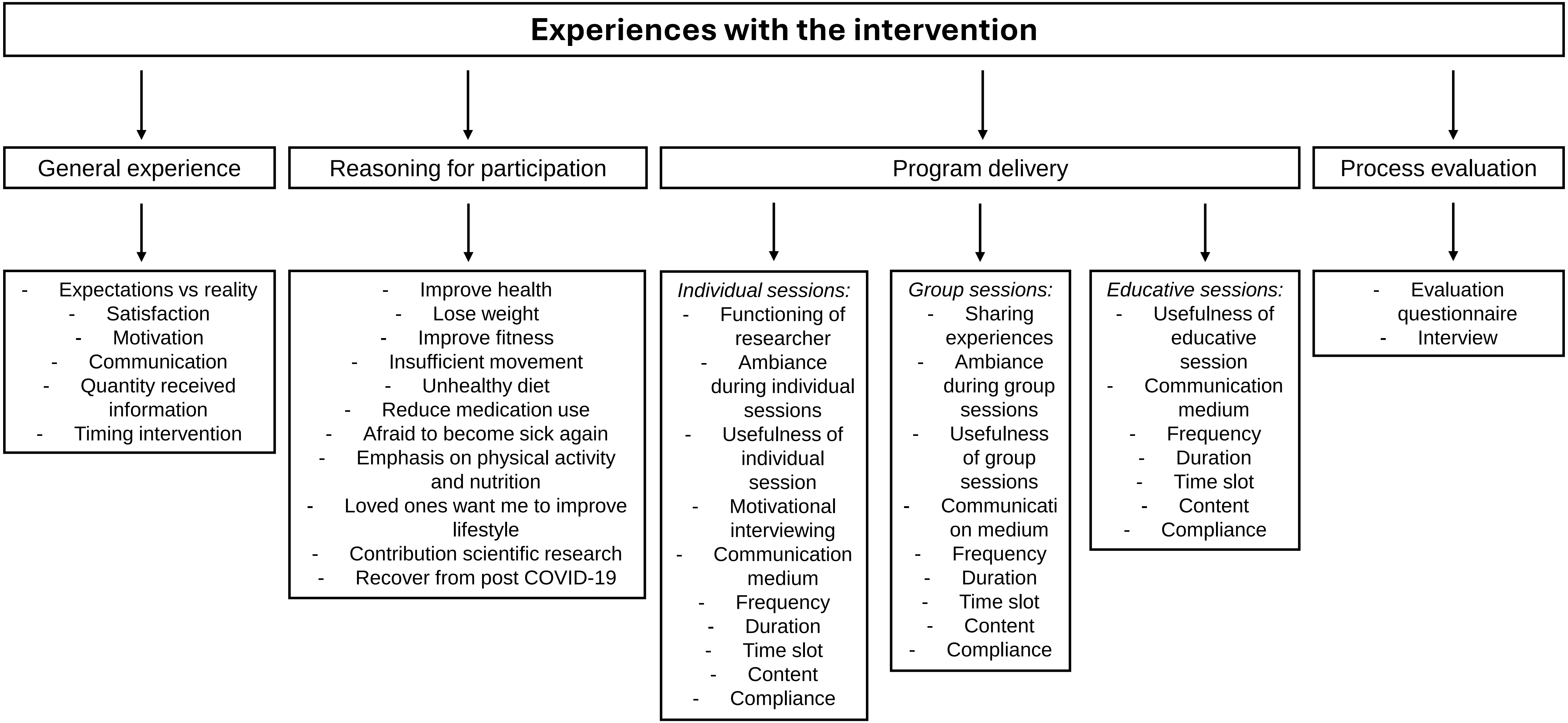
Experiences with the intervention
General experience
Reasoning for participation
Program delivery
Process evaluation
- Expectations vs reality
- Satisfaction
- Motivation
- Communication
- Quantity received information
- Timing intervention
- Improve health
- Lose weight
- Improve fitness
- Insufficient movement
- Unhealthy diet
- Reduce medication use
- Afraid to become sick again
- Emphasis on physical activity and nutrition
- Loved ones want me to improve lifestyle
- Contribution scientific research
- Recover from post COVID-19
Individual sessions:
- Functioning of researcher
- Ambiance during individual sessions
- Usefulness of individual session
- Motivational interviewing
- Communication medium
- Frequency
- Duration
- Time slot
- Content
- Compliance
Group sessions:
- Sharing experiences
- Ambiance during group sessions
- Usefulness of group sessions
- Communicati on medium
- Frequency
- Duration
- Time slot
- Content
- Compliance
Educative sessions:
- Usefulness of educative session
- Communication medium
- Frequency
- Duration
- Time slot
- Content
- Compliance
- Evaluation questionnaire
- Interview

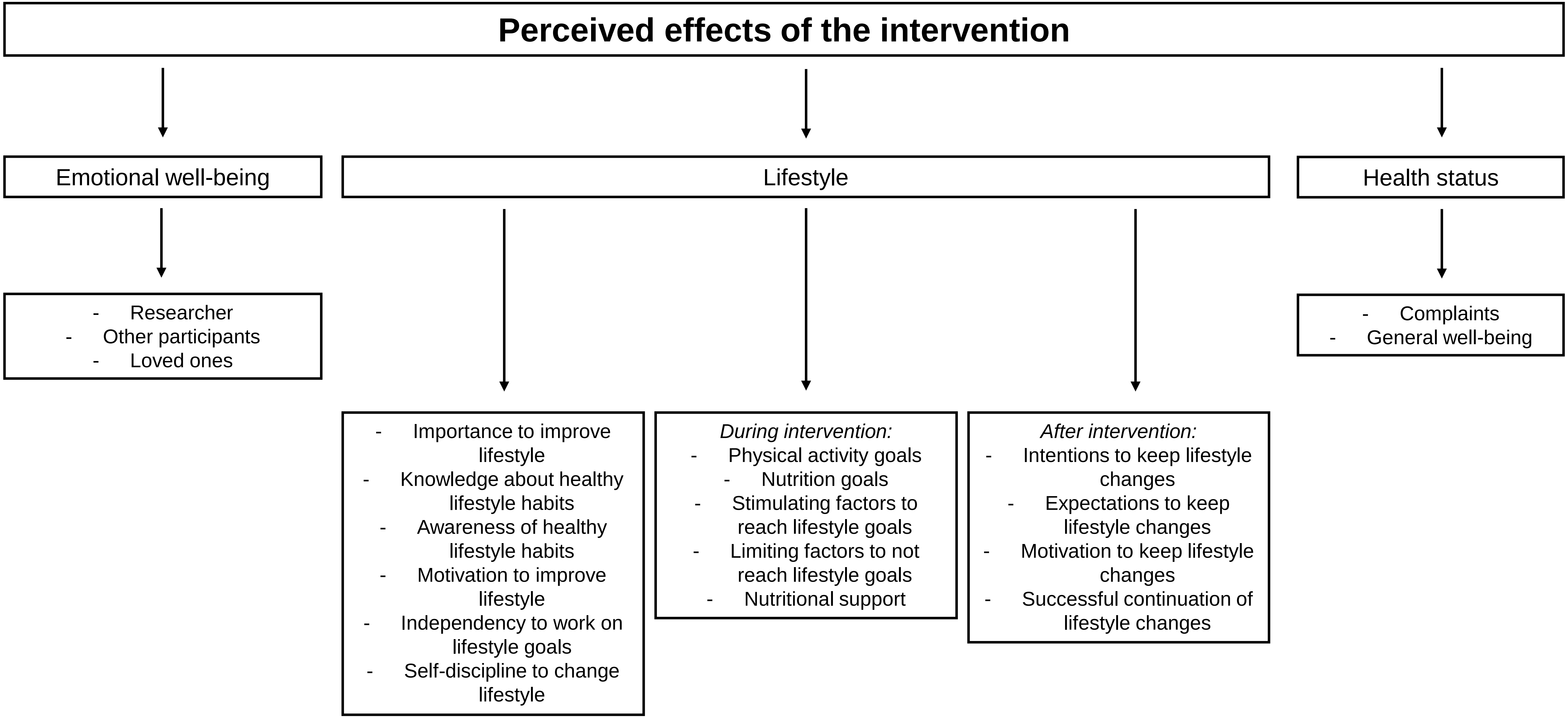
Perceived effects of the intervention
Emotional well-being
Lifestyle
Health status
- Researcher
- Other participants
- Loved ones
- Complaints
- General well-being
- Importance to improve lifestyle
- Knowledge about healthy lifestyle habits
- Awareness of healthy lifestyle habits
- Motivation to improve lifestyle
- Independency to work on lifestyle goals
- Self-discipline to change lifestyle
During intervention:
- Physical activity goals
- Nutrition goals
- Stimulating factors to reach lifestyle goals
- Limiting factors to not reach lifestyle goals
- Nutritional support
After intervention:
- Intentions to keep lifestyle changes
- Expectations to keep lifestyle changes
- Motivation to keep lifestyle changes
- Successful continuation of lifestyle changes
